# Morphine and high-fat diet differentially alter the gut microbiota composition and metabolic function in lean versus obese mice

**DOI:** 10.1038/s43705-022-00131-6

**Published:** 2022-08-05

**Authors:** J. Alfredo Blakeley-Ruiz, Carlee S. McClintock, Him K. Shrestha, Suresh Poudel, Zamin K. Yang, Richard J. Giannone, James J. Choo, Mircea Podar, Helen A. Baghdoyan, Ralph Lydic, Robert L. Hettich

**Affiliations:** 1 Genome Science and Technology Program, University of Tennessee, Knoxville, TN 37996 USA; 2 Biosciences Division, Oak Ridge National Laboratory, Oak Ridge, TN 37831 USA; 3 Pain Consultants of East Tennessee, PLLC, Knoxville, TN 37909 USA; 4 Department of Psychology, University of Tennessee, Knoxville, TN 37996 USA

**Keywords:** Microbiome, Symbiosis

## Abstract

There are known associations between opioids, obesity, and the gut microbiome, but the molecular connection/mediation of these relationships is not understood. To better clarify the interplay of physiological, genetic, and microbial factors, this study investigated the microbiome and host inflammatory responses to chronic opioid administration in genetically obese, diet-induced obese, and lean mice. Samples of feces, urine, colon tissue, and plasma were analyzed using targeted LC-MS/MS quantification of metabolites, immunoassays of inflammatory cytokine levels, genome-resolved metagenomics, and metaproteomics. Genetic obesity, diet-induced obesity, and morphine treatment in lean mice each showed increases in distinct inflammatory cytokines. Metagenomic assembly and binning uncovered over 400 novel gut bacterial genomes and species. Morphine administration impacted the microbiome’s composition and function, with the strongest effect observed in lean mice. This microbiome effect was less pronounced than either diet or genetically driven obesity. Based on inferred microbial physiology from the metaproteome datasets, a high-fat diet transitioned constituent microbes away from harvesting diet-derived nutrients and towards nutrients present in the host mucosal layer. Considered together, these results identified novel host-dependent phenotypes, differentiated the effects of genetic obesity versus diet induced obesity on gut microbiome composition and function, and showed that chronic morphine administration altered the gut microbiome.

## Introduction

Disruption of gut microbiota correlates with many negative health effects [1], including altered metabolism [2], pain [3, 4], and increased inflammation [5]. Obesity is prevalent in the western world [6] and is associated with disruptions in the gut microbiota [7]. Obesity also is associated with an increase in the abundance of inflammatory cytokines [8, 9] and chronic pain [10]. Obesity-triggered disruption of the microbiota manifests as a decrease in the overall richness/diversity of the gut microbiota and a decrease in the abundance of Bacteroidetes bacteria relative to Firmicutes bacteria [11–13]. Some of these findings, however, have proven difficult to reproduce in meta-analyses using body-mass-index (BMI) as the delimiter between lean and obese individuals [14–16]. These conflicting results may reflect the fact that obesity is a multi-dimensional disease. Different kinds of obesity affect the microbiome distinctly both in the magnitude of the effect and in the specific taxonomical changes in composition. This could explain some of these inconsistencies, as genetic obesity appears to increase the weight of mice more rapidly than high-fat diet, while high-fat-diet induced obesity appears to have a greater magnitude impact on the microbiota [17, 18].

Opioids, particularly morphine, are standard of care for treating chronic pain [19], and a recent study found that obese individuals are prescribed treatment with opioids at a higher rate than non-obese individuals [20]. Morphine and other opioids have been shown to alter the gut microbiome and induce a host inflammatory response in both mice and humans [21–24]. Mitigation of chronic pain with morphine may lead to undesirable perturbation of the gut microbiome as well as the host inflammation response [25]. Opioids, in particular morphine, have been shown to decrease the Bacteroidetes to Firmicutes ratio [21], decrease the abundance of bacteria from the family Lachnospiraceae [22], increase the species *Enterococcus faecalis* [23], increase cytokine IL-6 [22], increase bacteria associated with endotoxin [22], and disrupt the bile acid pool [21]. These studies, however, shared a common theme: opioids impacted the gut microbiota in a deleterious way regardless of oral [24] or subcutaneous [21, 23] administration, in both mice [21, 23] and humans [22, 24]. The investigation of the microbiota in each of these published studies was conducted exclusively with 16 S rRNA profiling, which limits the possible taxonomic resolution and functional understanding of gut microbiota effects. In addition, there does not appear to be any study that has quantified the effects of morphine relative to different kinds of obesity.

Given the overlapping epidemics of obesity and opioid abuse [26] and their association with altered microbial composition, the purpose of this study was to seek a better understanding of the effects of morphine on the gut microbiome and inflammation in the context of obesity. Mice provide a standard model for controlled manipulation of diet, genetic, and environmental variables enabling integrated omics analysis of microbial composition and function in a mammalian host [23, 27]. This study was designed to quantify alterations in the gut microbiota caused by chronic administration of an antinociceptive dose of morphine [28] to lean and obese mice. The gut microbiota were examined using a combined metagenomics and metaproteomics experimental design to provide increased taxonomic resolution and functional understanding of the study outcomes [29–34]. The goal was to understand the effects of continuously administered morphine on the gut microbiota at an antinociceptive dose that did not significantly alter mouse behavior.

## Results

**Fig. 1:**
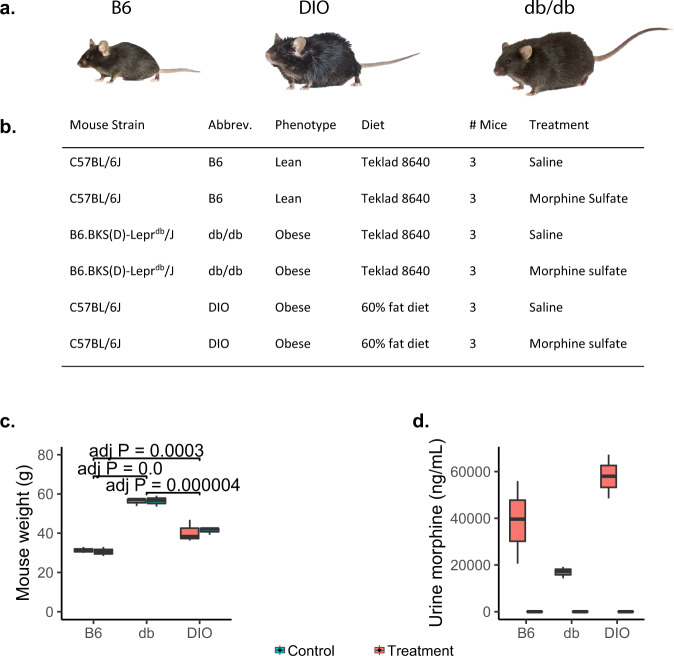
Experimental design. **a** Images depicting the phenotypic differences between the mice, printed with permission from © The Jackson Laboratory. **b** Table describing the different experimental groups. All measurements were conducted on all mice. **c** Boxplot depicting weight of mice at time of sample collection. *P* values calculated by TukeyHSD, only a single significant digit reported. **d** Boxplot depicting morphine quantities in mouse urine analyzed by triple quadrupole mass spectrometer.

Three groups of mice (18 total) sharing a C57BL/6 J (B6) background were used to compare gut microbiota changes due to morphine (Fig. 1a, b). The control group comprised B6 mice (*n* = 6) fed a standard mouse diet. A second group of mice were B6 mice with diet-induced obesity (DIO; *n* = 6) caused by consuming a 60% fat diet. The third group comprised B6.BKS(D)-LepR^db^/J mice (db/db mice, *n* = 6) fed a normal diet yet were obese due to a spontaneous mutation of the leptin receptor. The db/db mutation confers susceptibility to obesity and type-2 diabetes through a muted satiety-response [35]. Three mice from each of the three lines were treated either with saline (vehicle control) (*n* = 9), or morphine administered continuously for two weeks via implanted ALZET pumps (*n* = 9). The three mouse groups had different weights, with db/db mice heavier than DIO, and DIO heavier than B6 mice, as has been previously noted (Fig. 1c, Supplementary Table S1) [18]. Morphine-derived metabolites were detected only in mice treated with morphine, with the db/db mice having significantly lower levels of such metabolites (Fig. 1d, Supplementary Table S1). Microbiome samples were collected by extracting intact feces directly from the colon via surgery.

### Comprehensive metagenomic analysis of mouse gut microbiota reveals mostly previously unknown microbes

**Fig. 2:**
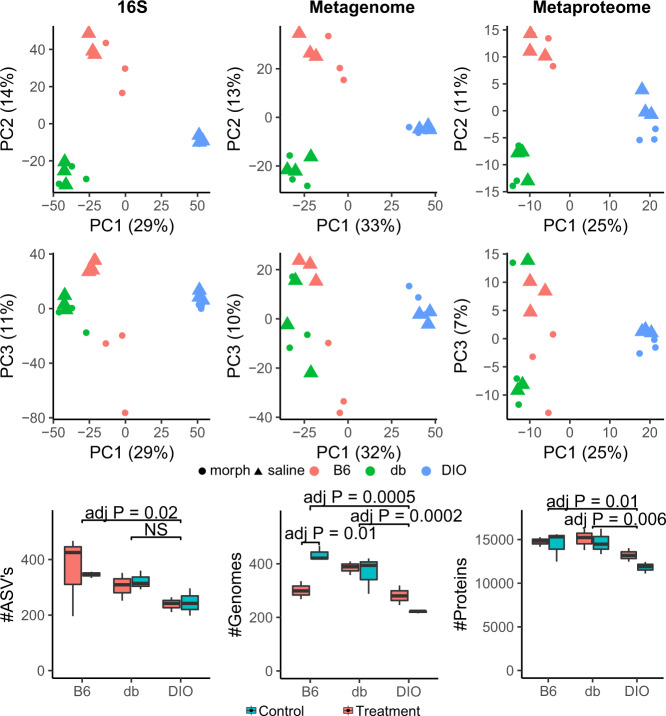
Diet-induced obesity caused the greatest variation in microbiome composition, while morphine effects were evident only within the B6 line. PCA analysis of normalized abundance of the 16 S ASVs and non-redundant metagenome assembled genomes. Metagenome abundances were calculated by inner-quantile mean coverage and summed spectral counts of unambiguously assigned proteins. Box plots depict number of ASVs, non-redundant genomes, and proteins quantifiable in each sample. *P* values were calculated by Tukey HSD after two-way ANOVA comparing both line and treatment, only a single significant digit reported.

To obtain an overview of the mouse gut microbiome’s composition and guide subsequent whole genome metagenomic sequencing, 16 S rRNA amplicons derived from feces from all mice in Fig. 1b table were sequenced to saturation revealing 196-468 amplicon sequence variants (ASV) per sample, totaling 1197 ASVs across all samples (Fig. 2; Supplementary Table S2). Matching ASVs to the Silva rRNA database revealed that 95% of the ASVs could not be classified to the species level [36]. This indicates that many of the mouse gut bacteria are not represented in the common sequence repositories, suggesting a need for metagenomic analysis prior to metaproteomics.

Metagenomic sequencing of each colon-extracted microbiome sample resulted in at least 250 million reads per sample (2 × 150 bp). Sequence binning and curation extracted 590 medium-quality metagenome-assembled genomes (MAGs) (Supplementary Table S3) [37]. Between 215-467 genomes were detected above the quantification threshold per sample, suggesting a comprehensive extraction of MAGs from the metagenome based on the 16 S results (Fig. 2; Supplementary Table S3). The MAGs represent 460 distinct species with a 95% ANI threshold, which has been shown to be an appropriate species cutoff across several studies [38–40]. Only 7% of the genomes were represented in the Genome Taxonomy Database, which is compiled from RefSeq and GenBank [41, 42]. As the number of named species assembled was even lower, the mouse microbiome remains underrepresented in sequence databases. Supporting this, BLAST searches of all MAG-derived protein sequences against the UniParc, NR databases, and a mouse catalog yielded mean top BLAST alignments < 90% amino acid identity (Supplementary Fig. 1c) [43–45].

Predicted open reading frames from the MAGs were used to create the reference database for the metaproteomic analysis. In total, 1,212,277 non-redundant protein sequences were derived from the MAGs, and these were combined with murine host proteins to generate a protein sequence database for searching MS/MS peptide spectra. 97% of these non-redundant proteins were resolvable at the species level. This enabled the identification and subsequent quantification of gene-products/proteins derived from previously uncharacterized species. Across all samples and conditions, a total of 115,628 proteins were detected with at least one protein unique peptide, with approximately 11,000-16,000 non-redundant proteins detected per sample (Fig. 2; Supplementary Table S4). Less than 10% of the proteins detected in each sample belonged to the host; the remainder were bacterial in origin. Approximately 70% of the identified proteins were assigned a functional annotation using the eggNOG database [46], enabling subsequent metabolic interrogation across the mice and treatments.

### Morphine alters the gut microbiome’s composition but obesity eclipses the effects of morphine

To evaluate high-level changes in microbial composition between conditions directly by the three omics approaches, mean-centered log transformations of ASV read frequency and genome abundance—calculated by metagenomic coverage and metaproteomic abundances (spectral counts)—were evaluated by principal component analysis (Fig. 2). Abundances derived from all three omic platforms showed the same data profile. Microbial composition was affected most by diet induced obesity, which separates along the first component (25–33% of the variance). The second greatest effect was observed in db/db mice separating along the second component (11–14% of the variance). Morphine was shown to have a lesser effect, separating along the third component, but only in the lean mice (7–11% of the variance). Hierarchical clustering of 16 S and metagenomic data showed similar results: (1) DIO separated from db/db and B6, (2) db/db separated from B6, and (3) B6 treated with morphine separated from B6 treated with saline (Supplementary Fig. S1a, b). There were no significant phylum level differences between the conditions (Supplementary Fig. S2).

To determine if morphine or obesity-type affected the diversity of gut microbial communities, 16 S data was evaluated using alpha diversity metrics. No metric of alpha diversity indicated any differences in sample diversity between conditions (Supplementary Fig. S3). The total number of ASVs, genomes, and proteins detected in each sample was significantly lower in DIO, suggesting lower community richness imparted by the high-fat-diet – results that are in agreement with a recent meta-analysis (Fig. 2) [47]. Given that db/db mice have a significantly greater weight than DIO mice (Fig. 1c), this suggests that diet drives the observed decreases in microbiome complexity and is not a result of obesity as a generic class. Fewer quantifiable genomes were detected in morphine treated B6 mice relative to saline-treated B6 lean mice (Tukey HSD adjusted *P* = 0.01) (Fig. 2); however, this effect was not observed in the obese mice nor with regards to the number of ASVs or proteins identified.

### Metaproteomic analysis reveals that taxa-level protein abundances were specific to sample conditions

**Fig. 3:**
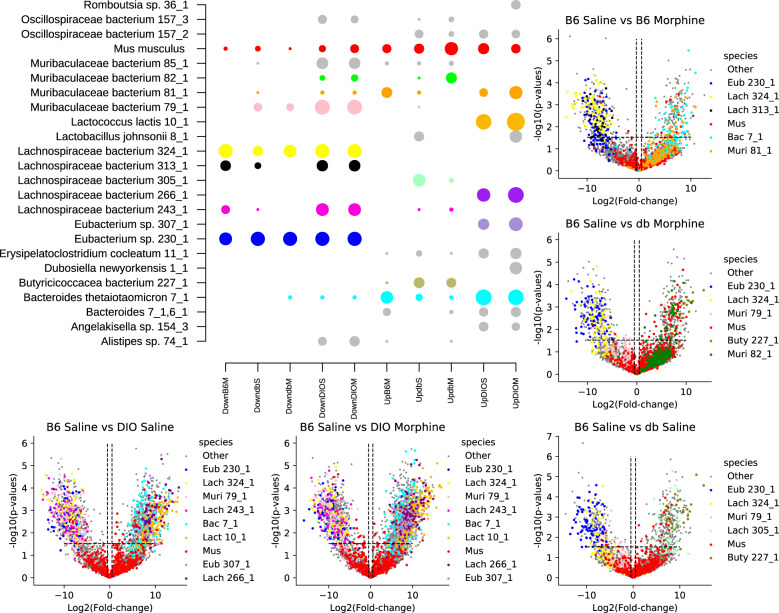
Differential protein abundance analysis reveals specific Lachnospiraceae species that favor B6 saline over B6 morphine and are distinct for different kinds of obesity. Bubble plot of species with at least 15 or more significant proteins in a comparison between B6 saline and other experimental conditions. Volcano plots of each binary comparison among B6 saline and another experimental condition. Dotted black lines depict a log_2_ fold change of 0.5 or greater, and Benjamini-Hochberg procedure Q < 0.1 threshold. *P* values were calculated by student *t* test. Colors are species specific.

To decipher details behind changes in microbiome composition correlated with morphine treatment or obesity-type, data from the metaproteomic measurement were evaluated for proteins that were significantly different among sample groups. Across all samples, there were 7324 proteins identified in at least three of the replicates of a condition. Of these, 3208 exhibited differential abundance relative to the B6 saline control. In almost all cases, these differentially abundant proteins were present in all three replicates of one condition and absent from all three replicates in one or more other conditions (Supplementary Table S5). Changes in relative protein abundances between the B6 saline control versus a given sample type reflected changes in microbial species composition rather than shifts in community function, which explains why differentially abundant proteins were more driven by presence versus absence rather than quantitative differences. Species enriched among differential proteins trended with the number of proteins detected for that species, which likewise aligned with the genome coverage of that species (Fig. 3; Supplementary Fig. S4). Thus, the species classification of differential proteins may be a good signal for differential abundant species in a condition. In this vein, specific species from the Lachnospiraceae family (labeled *Eubacterium* sp. or *Lachnospiraceae* *bacterium* for new genera) were enriched among proteins that were decreased in morphine treated B6 mice, db/db mice, and DIO mice relative to B6 mice treated with the saline control (Fig. 3). This aligned with a LefSe [48, 49] analysis of the 16 S data, which indicated that the Lachnospiraceae family was reduced in all conditions relative to the B6 saline mice. (Supplementary Fig. S1d).

### Both diet and morphine alter microbiome metabolic function in specific and distinct ways

**Fig. 4:**
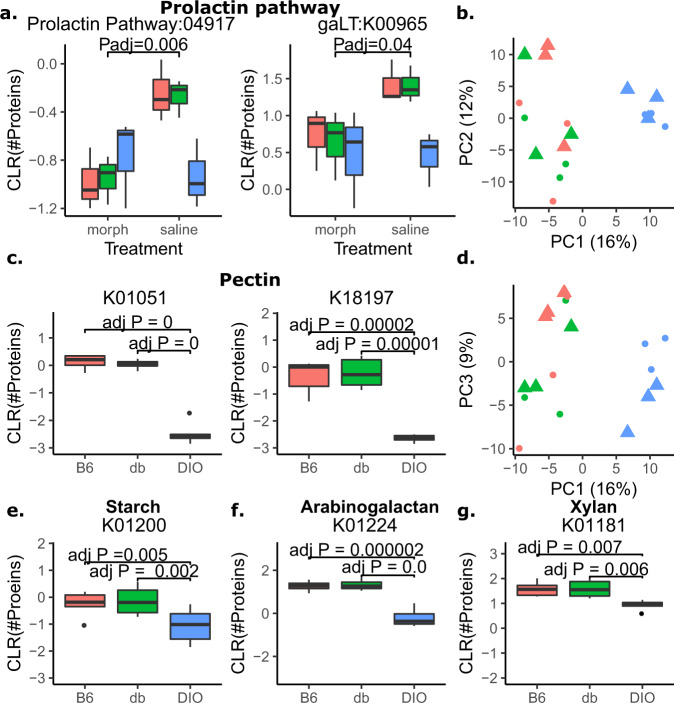
Total proteins in functional categories reveals functional shifts between morphine versus saline and B6 and db/db versus DIO mice. **a** Boxplots depicting the difference in relative number of proteins between morphine and saline treated mice in the prolactin pathway and its constituent enzyme gaLT. **b**, **d** The first, second and third components of principal component analysis of the number of detected proteins for each KEGG ortholog. **c** Representative boxplots of the relative number of proteins between lines for KO terms associated with pectin degradation. **e**–**g** Representative boxplots of relative number of proteins between lines of KO terms associated with starch, arabinogalactan, and xylan degradation. All boxplots were significant by two-way ANOVA (Q < 0.25) and TukeyHSD adjusted *P* value < 0.05, only a single significant digit reported.

Since protein enumeration aligns with species abundance, it was inferred that this would apply to functional classifications as well. To explore changes in overall microbial function between conditions, the number of proteins mapping to specific KEGG modules/pathways (Supplementary Table S6) and KEGG orthologs (Supplementary Table S7) was tabulated [50]. PCA analysis of counted proteins for KEGG orthologs revealed that regardless of treatment, DIO mice were distinct from db/db and B6 mice via the first principal component, while B6 and db/db mice did not separate at all, indicating that differences in microbial function between lean and obese mice are likely driven by diet (Fig. 4b, d). This same PCA analysis did not reveal any overall functional effects between mice treated with morphine versus saline either between mice from all lines nor mice within the B6 line (Fig. 4b, d).

Despite this result, three KEGG pathways/modules had a significantly different relative number of proteins between morphine- and saline-treated mice for all lines. The prolactin signaling pathway (ko04917) (Fig. 4a) and the nucleotide sugar biosynthesis module (M00554) were both over-represented in saline-treated mice relative to morphine-treated mice, while an ascorbate biosynthesis module (M00129) was over-represented in morphine treated mice relative to saline treated mice (Supplementary Table 6).

Forty-one KEGG pathways/modules were found to be significantly different between mouse lines. Of these, 31 trended similar for db/db and B6 mice. These pathways include pectin degradation, pentose and glucuronide interconversions, ascorbate and alderate metabolism, and methanogenesis. Conversely, glutathione metabolism, aminobenzoate degradation, fat digestion and absorption, and thyroid hormone synthesis pathways were all observed to be increased in DIO than in db/db and B6 mice (Supplementary Table S6).

A similar analysis conducted for KEGG orthologous groups revealed that most of the significant orthologs were driven by a few proteins detected in one condition and not detected in another, a finding which is not reflective of a bulk microbiome transition (Supplementary Table S7). Despite this, a couple of interesting orthologs stood out. Glutathione reductase, K00383, was increased in DIO mice relative to B6 and db/db mice. Glutathione reductase helps the microorganism survive oxidative stress [51] shown to be induced by consuming a high-fat diet [52]. Another interesting ortholog was the large conductance mechano-sensitive channel, K03282, which protects cells against extreme turgor [53]. This ortholog was significantly increased in morphine relative to saline treatments across all mouse lines revealed by two-way ANOVA, and the difference between morphine and saline was most obvious in the B6 and db/db lines.

To better observe the larger scale and most robust transitions in microbiome function, low count KEGG orthologs (KO) were removed prior to re-analysis. KEGG orthologs that showed significant differences in representation between mouse lines further substantiated the close relationship between db/db and B6 mice (Supplementary Table S8). Several of the KO terms that were significantly increased in db/db and B6 relative to DIO consisted of enzymes that release sugars from known plant fibers, including pectin, starch, arabinogalactan, and xylan (Fig. 4c, e-g) (Supplementary Table S8).

**Fig. 5:**
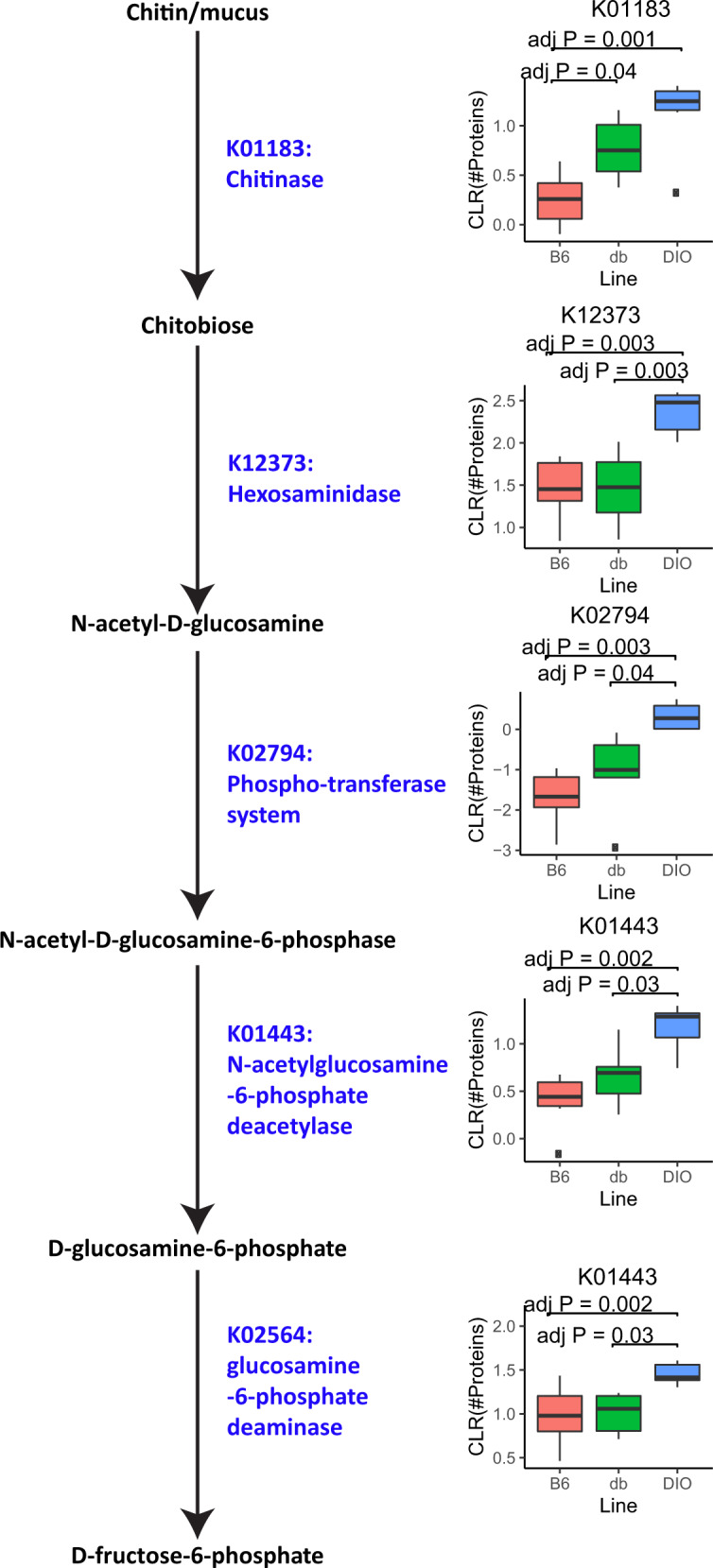
Total proteins in KEGG orthologs reveals a complete pathway from mucus to D-Fructose 6-phosphate that is more abundant in DIO than B6 or db/db mice. Proposed pathway from mucin to fructose-6-phosphate supported by boxplots of CLR normalized protein counts of KEGG orthologs associated with the pathway. All KO terms were significant with a TukeyHSD adjusted *P* value < 0.05 between DIO and B6 and/or db/db. All KO terms, except K02564 were significant by two-way ANOVA (Q < 0.25). Only a single significant digit was reported.

In contrast, KO terms over-represented in DIO mice relative to B6 and/or db/db mice suggested that a high-fat diet triggers starvation stress in the microbial community and transitions the microbiome away from consuming dietary inputs towards foraging host mucous for nutrients. Trimeric autotransporter adhesin and flagellar assembly factor, virulence factors involved in host cell invasion and adherence to components of the extracellular matrix such as glycoproteins [54, 55], as well as spoIIIAA, a protein critical for initiation of sporulation [56], each had significant increased representation in DIO than db/db mice and/or B6 mice (Supplementary Fig. S5). Many DIO-responsive orthologs mapped to the KEGG amino sugar and nucleotide sugar pathway, which contains enzymes that catalyze fucose, acetylneuraminate, and acetylglucosamine (Fig. 5; Supplementary Fig. S5). Mucins make up the majority of the intestinal mucosal layer, and mucins’ serine and threonine residues connect to glycans made up of fucose, acetylneuraminate, and acetylglucosamine, along with acetylgalactosamine and galactose [57]. Acetylgalactosamine and galactose catalyzing enzymes were not observed to be significantly different between mouse lines.

Several glycoside hydrolases, specifically hexosaminidase, fucosidase, galactosidase, and sialidase, release the different components of mucin glycans [58]. Galactosidases, though present, did not appear to be differentially abundant between conditions; however, a sialidase ortholog, K01186, and a fucosidase ortholog, K01206, were each increased in DIO versus B6 or db/db, respectively. Enzymes that subsequently catalyze fucose and acetylneuraminate reactions were over-represented in DIO as well, specifically L-Fucose/D-arabinose isomerase and N-acetylneuraminate lyase (Supplementary Fig. S5). The hexosaminidase KEGG ortholog K12373 was significantly increased in DIO relative to B6 and db/db mice, which releases acetylglucosamine from glycans. Additionally, the chitinase ortholog K01183, which also releases acetylglucosamine from glycans, specifically chitin, was significantly increased in DIO and db/db relative to B6 mice. In addition, all of the enzymes needed to connect acetylglucosamine to fructose-6-phosphate (glycolysis) were present in the samples and were overrepresented in the DIO mice (Fig. 5). These enzymes were organized into a pathway as follows: release of acetylglucosamine from mucin via chitinase, hexosaminidase, and xaa-pro dipeptidase orthologs, followed by the import of acetylglucosamine into the cell by phosphotransferase system orthologs, the deacetylation of now acetylglucosamine-6-phospate by N-acetylglucosamine-6-phosphate deacetylase orthologs, and the conversion of glucosamine-6-phosphate to fructose-6-phospate by glucosamine 6-phosphate deaminase orthologs. The evidence for all of these orthologs came from a myriad of species across the different samples, and all were over-represented in DIO relative to db/db and B6 mice, with two caveats. The PTS system detected was actually the mannose PTS system EIIAB component; however, this system has been shown to also import acetylglucosamine [59]. The final enzyme described here, glucosamine 6-phosphate deaminase, was not significant by Q value, but was significant by *P* value (*P* = 0.01).

## Distinct inflammatory signals correlate with obesity and morphine treatment

To determine the inflammatory signal caused by morphine, high fat diet, or genetic obesity, plasma and protein derived from colon tissue samples were analyzed by a magnetic bead panel of mouse chemokines and cytokines (Supplementary Table S1). Most cytokines were not detectable, but KC (Mouse IL-8), TNF-α, and IL-6 were more abundant in plasma of db/db relative to B6, DIO relative to B6, and B6 treated with morphine relative to B6 treated with saline control respectively (Supplementary Fig. S6). Eotaxin a chemokine associated with the healthy development of the mucosal layer [60] was depressed in both db/db and DIO colon tissue relative to B6 colon tissue, providing evidence for dysbiosis.

## Discussion

This study shows that an antinociceptive dose of morphine administered continuously for two weeks via a subcutaneous osmotic pump altered the composition of the gut microbiota in mice. PCA analysis and hierarchical clustering of 16 S rRNA, metagenomic, and metaproteomic data revealed notable changes in the lean, wild type mice. Previous studies in mice and humans across multiple opioids and delivery methods (subcutaneous pellets and oral doses) have indicated that opioids alter the microbiome as well [21–24]. Those studies, which were all limited to 16 S rRNA profiling, and highlighted different organisms and microbiota features as indicators of the effect of morphine on the gut microbiota. Interestingly, the present results align most closely to the results from the human studies.

The present proteomic, metagenomic, and 16 S rRNA results indicated that specific species from the Lachnospiracea family were suppressed by morphine [22]. In addition, the number of genomes with at least 2X coverage was reduced in morphine-treated B6 mice relative saline-treated B6 mice. This effect was not observed in the proteomics or 16 S data. This decrease in richness aligns with the results of two of the above studies in mice [23] and humans [24]. The similarities in morphine effect supports the interpretation that some opioid effects on mouse microbiota may be translatable to human.

There was minimal effect of morphine treatment on the gut microbiota in mice with obesity resulting from diet or genetics. This finding suggests that the inherent dysbiotic effect induced by either type of obesity obscures the effect of morphine on the gut microbiota. In addition, the two types of obesity each had distinct gut microbiota compositions, as observed in previous studies [17, 18]. Morphine, db/db, and DIO treatments each knocked down the same or similar species from the Lachnospiracea family, though in many cases they were replaced by different species in each group. The decreased species richness of microbiota was more consistently associated with diet induced obesity than with morphine administration. Interestingly, the effect of these two obesity types on specific species from the Lachnospiracea family did not apply to all microbial members. For example, *Lachnospiraceae bacterium 266_1* and *Eubacterium sp. 307_1* were more abundant in DIO mice relative to B6 mice in the saline condition.

Distinct from taxonomic changes, overall alterations in microbial function due to the experimental conditions were analyzed by tabulating the number of proteins assigned (with normalization) to a KO term, pathway, or module. As opposed to taxonomic composition, no obvious changes in microbial function were observed by PCA analysis due to db/db-induced obesity or morphine, while diet induced obesity had a distinct functional profile. Despite this, some specific functional changes were observed due to morphine treatment. In particular, the KEGG prolactin pathway had significantly more protein representation in saline-treated mice than in morphine-treated mice. This observed increase of the prolactin signaling pathway in saline relative to morphine was surprising because morphine promotes the production of prolactin [61]. Interestingly, this result was driven by microbial orthologs of the protein gaLT (Fig. 4a) (K00965), which is suppressed by prolactin in the host [62]. This suggests host hormones may also suppress the microbial orthologs of their host targets.

The KEGG ortholog most significantly increased by morphine was the large conductance mechano-sensitive channel, K03282, which protects cells against extreme turgor [53]. Though there could potentially be other explanations, one of the side effects of morphine is to cause increased urinary retention, which in extreme cases can lead to low serum osmolality (hyponatremia), resulting in an excess water relative to solute [63–65]. These relationships, along with the prolactin results, suggests that known impacts of morphine on the host may affect microbial functionality as well.

While a few studies have used metaproteomics to explore the effects of obesity on the microbiome [66–69], we are not aware of any previous study that has used metaproteomics to compare diet versus genetic-driven obesity phenotypes directly. Our results revealed that genetically obese (db/db) and lean B6 mice were quite similar with regards to KEGG function, while the diet-induced obesity (DIO) mice were distinct, implying that a change in microbial function is more strongly driven by diet than obesity as a generic category.

This high-fat diet-induced change in microbial metabolic function manifested as a decrease in dietary fiber-degrading enzymes, and an increase in enzymes associated with the metabolism of acetylglucosamine, sialic acid, and fucose, which are components of mucin (Figs. 4, 5; Supplementary Fig. S5). This diet decreased the availability of fibers, in addition to other nutrients, in exchange for fat. This decrease in dietary carbohydrates likely would lead to starvation-induced stress and push gut microbes toward foraging the mucosal layer for sugar. Increased microbial forging of the host mucosal layer due to poor nutrition has been demonstrated for low-fiber and westernized diets in several studies [70–73]. Though it has been suggested that the capacity for mucosal foraging may be widespread, these studies for the most part are species specific. In particular, *Bacteroides thetaiotaomicron* and *Akkermansia muciniphila* have been shown to forage the mucosal layer for nutrients [72, 73]. Both *Bacteroides thetaiotaomicron* and *Akkermansia muciniphila* were detected in this study, and several of the orthologs described above were detected for these species, but they were not the primary factor for the observation that a high-fat, low fiber diet drives the microbiome towards host foraging. Instead, a novel aspect of the present results is evidence that this transition was not driven by a specific subgroup of species, but by a variety of species from different phylogenetic groupings. For example, distinct species (n) had protein evidence for hexosaminidase (*n* = 43), chitinase (*n* = 47), phosphotransferase system (*n* = 16), n-acetylglucosamine-6-phosphate deacetylase (*n* = 71), and glucosamine 6-phosphate deaminase (*n* = 97). An important component of the pathways described above is the incorporation of free acetylglucosamine into the cell prior to its involvement in central metabolism via glycolysis. This suggests that acetylglucosamine could be cleaved from mucus by one species and used for energy by another. These data indicate a community-driven, rather than species-driven, transition in function. This functional shift is not apparent when focusing on individual proteins or species and is instead indicative of community aggregate function.

The majority of the functional effects observed in this study were metabolic, so it may be no surprise that diet induced obesity stood out has having the greatest functional effect on the microbiome. It is possible that higher dosages or an oral delivery route could have yielded additional or different functional changes in the microbiome or observable taxonomic differences in the obese mice due to morphine. Additionally, future studies using metabolomics on serum or feces could provide further insights by connecting observed metabolites to the proteins detected in this study. The focus of this study and thus the samples that were targeted were designed to capture the holistic functional potential of the colon microbiome; thus, future studies on more localized samples taken from the cecum or specific locations in the colon might reveal distinct spatial effects.

There are three features of the experimental design that were included to reduce variability rather than to emulate pain medicine. First, the results are from only male mice although the gut microbiome is known to be sexually dimorphic [74]. The present study focused on male mice because obesity caused by a high fat diet is sexually dimorphic [75] such that female B6 mice gain less weight than male B6 mice [76]. Second, the administration of morphine in the absence of acute or chronic nociception does not emulate clinical scenarios. A third feature is the extent to which the present results would be altered by periodic opioid administration, rather that tonic infusion of morphine. Despite these factors, the results encourage future studies designed to include microbiome measures before and after administering opioids that diminishes experimentally induced nociception.

As a special note, many of the genomes uncovered by this study were not present in the Genome Taxonomy Database, which is representative of NCBI databases [41, 42], and further analysis of the genes derived from these genomes indicated that they were not present in either the NCBI databases [43] nor UniProt [77]. This is in line with recent investigations of the mouse gut microbiome, which simply implies that the mouse gut microbiome is not well represented in these databases despite the extensive research that has been done on this system [78, 79]. These genomes have been submitted to NCBI (Supplementary table 3), as such providing a resource for future studies of the mouse gut microbiome.

Considered together, the present results show that an observable change in microbial *composition* does not necessarily translate to an observable change in microbiome *function*. Morphine, genetic obesity, and high-fat diet consumption all had an impact on total microbial composition, but a clear functional shift was only observed among mice fed a high-fat diet. The results suggests that caution should be exercised when attributing a microbiome change to specific taxonomic features and highlights the importance of looking at microbiome function concurrently with taxonomic composition, as the former tends to be more stable [33, 80]. In tandem with the distinct changes in the gut microbiota composition and function shown here, distinct host physiological effects were observed as well. Each variable tested was observed to have distinct inflammatory cytokines increased in abundance relative to the lean control. As previously observed, morphine caused an increased abundance of IL-6 in the lean mice [22] and both DIO and db/db mice revealed a significant decrease in eotaxin in their colon tissue. Eotaxin is associated with the production of mucin [60], providing physiological evidence for the dysbiosis associated with a disrupted microbiome.

## Methods

### Study design and sample collection

The protocol for these studies was reviewed and approved by the Institutional Animal Care and Use Committee of the University of Tennessee. Mouse numbers, ample size, and route of drug administration followed the ARRIVE guidelines [81]. Adult male wild-type mice C57BL/6 J (B6; Stock No: 000664; *n* = 6), B6 mice with diet-induced obesity (DIO) (Stock No: 380050; *n* = 6), and obese mice with a spontaneous mutation of the leptin receptor B6.BKS(D)-*Lep*^db^/J (db/db; Stock No: 000697; n = 6), were purchased from The Jackson Laboratory (Bar Harbor, ME). The B6 and db/db mice were fed Teklad 8640 rodent chow containing 5% fat (Envigo, Madison, WI). The DIO mice were fed a 60% fat diet (Research Diets catalog number D12492). Thus, mouse genotype and sex were held constant and obesity was a subject variable. Environmental housing conditions for all mice included a 12:12 light:dark cycle, temperature and humidity control, and free access to food and water.

Mice were weighed, anesthetized with isoflurane, and implanted with Alzet osmotic pumps delivering 10 mg/kg/day of morphine sulfate (Sigma Chemical, Cat. No. M8777; independent variable) at a rate of 0.5 uL/h, or saline (vehicle control) for two weeks. The 10 mg/kg dose of morphine was chosen because it is a dose that is known to be antinociceptive in wildtype B6 mice [28]. After two weeks, urine samples were used to confirm morphine metabolism using multiple reaction monitoring on a Shimadzu LCMS-8040, and all mice were anesthetized with isoflurane and terminal samples (blood plasma, colon tissue, and fecal material extracted from colon) were obtained from all 18 mice. Terminal blood was collected from right ventricle in EDTA-treated collection tubes then centrifuged at 3000 rpm. Collected plasma, colon tissue, and fecal material were frozen immediately at –80 ^o^C.

### Cytokine analysis

Cytokines were quantified in plasma and colon tissue by Milliplex MAP Mouse Cytokine/Chemokine Magnetic Bead Panel (Millipore Sigma, Burlington, MA) premixed 32-plex kit according to the protocol. Exploratory data analysis using PLS-DA in the MixOmics R package was used to predict which cytokines were significantly more abundant in each line, and in morphine treatment [82]. Two-way ANOVA followed by Tukey HSD was used to confirm the prediction.

### Metagenomic and amplicon sequencing

DNA extraction was performed using a Quick-DNA Fecal/Soil Microbe kit (Zymo Research, Irvine CA), according to manufacturer’s protocol on remaining lysate from protein extraction method. The extracted DNA was further purified using the DNA Clean & Concentrator kit (Zymo Research) and quantified by fluorometry using a Qubit assay (ThermoFisher Scientific). Metagenomic sequencing was performed at the Sequencing Center of HudsonAlpha Institute (Huntsville, AL). Genomic DNA from 18 colon fecal samples was sheared and used to generate libraries that were sequenced (2 × 150 nt) on an Illumina NovaSeq instrument. The raw reads quality checked and trimmed using Trim Galore (v. 0.6.0) https://www.bioinformatics.babraham.ac.uk/projects/trim_galore/. Reads were then assembled with an aim to extract metagenome assembled genomes using a modified version of the procedure described in Olm et al. [83]. In brief, all 18 samples where co-assembled using MEGAHIT (1.1.3) with the parameters k-min set to 31 and k-step set to 10 [84]. Mouse and human DNA was removed using bbsplit (v 38.31). Each sample was independently assembled using idba_ud (1.1.3) [85]. Reads from all samples were mapped to all assemblies using bwa (v. 0.7.17-r1188) piped to samtools (v. 1.9) creating sorted bam files [86, 87]. Assemblies were then binned using Metabat2 (v. 1.12.1) [88] and bins were quality checked using checkM (v. 1.1.2) [89]. Bins greater than 50% complete and less than 10% contaminated were considered medium quality [37]. Bins that did not pass initial threshold were manually curated by tetranucleotide frequency using Anvi’o (v. 5.2.0) [90]. All medium quality genomes, were dereplicated using dRep (v. 2.2.3) at 99.5% ANI and 95% ANI to create non-redundant genome and species clusters, with a representative genome selected for each cluster [83]. Protein sequences from the assemblies were extracted from the checkM result and annotated using eggNOG-mapper (v. 1.1.0.3-40-g41a8498) [46, 91]. Abundance for each representative genome was calculated using the inner-quantile mean coverage (Q2Q3 mean coverage) acquired from Anvi’o.

For 16 S rRNA amplicon sequencing, the V4 hypervariable region of the 16 S rRNA gene was amplified using universal primers derived from 515 F and 806 R primers [92] fused to Illumina sequencing adapters, following the procedure developed by [93]. The final amplicons were pooled, purified using Agencourt Ampure XP beads, quantified by Qubit, and sequenced (2 × 250 nt) on an Illumina MiSeq instrument (Illumina Inc, San Diego, CA) using a v2 500 cycle kit. Raw sequence reads were trimmed of the PCR primers/adaptors using cutadapt (10.14806/ej.17.1.200) and joined using the QIIME script join_paired_ends.py. Joined reads were processed and analyzed using the QIIME2 (v. 2019.1.0) and MicrobiomeAnalyst [94, 95]. Taxonomic assignment was performed against the SILVA SSU rRNA database (v. 132) [36, 96].

### Metaproteomic identification LC-MS/MS

Colon extracted fecal samples were lysed with bead-beating in SDS buffer, treated with 25 mM DTT and 75 mM IAA. Protein was precipitated using a chloroform methanol extraction protocol and digested with trypsin. Peptide was quantified using bicinchoninic acid (BCA) assay and 12 ug of peptide for each sample was loaded onto a two-dimensional chromatography system made up of a strong cation exchange back column and a 30 cm C18 analytical column with an electrospray emitter. Peptides were separated across 4 salt pulses (35, 50, 100, 500 mM ammonium acetate) and 150-minute reverse phase gradient and measured on a Q-Exactive Plus mass spectrometer. Peptides were detected using data dependent acquisition, with 5 peptides isolated for MS2 HCD fragmentation per MS1. Detected mass-to-charge ratios were placed on a 30-minute exclusion list. Raw data was collected using Xcalibur (v. 4.1.31.9) and converted to mzML using msconvert (v. 4.1.31.9) [97]. A protein database was constructed from the representative genomes from the metagenome, a list of common protein contaminants, the human reference proteome. This protein database was clustered at a 98% identity to remove redundancy using CD-HIT [98, 99]. The 98% cutoff was selected as it was the minimum cutoff at which there was minimal loss of species specificity. Mass spectra were search against this database using a two-step search via the crux (v. 3.2.8aa66a2) toolkit (tide-search with exact p-value calculation and percolator for FDR calculation) [100–102] (see supplementary method for details). For a protein to be identified, it needed to have at least one unique peptide identified with a peptide spectral match FDR of less than 1%. Protein abundance for each sample was determined by MS1 apex intensity, using moFF (v. 2.0.3) [103]. Protein abundance was log2 transformed, LOESS normalized, and mean centered using Inferno (v. 1.1.7234) [104]. Normalized proteins intensities were filtered down to proteins that were quantified at least 3 times in a condition. These proteins were then submitted to serial t-tests between B6 control, and each other experimental condition (B6 T, db/db S, db/db T, DIO S, DIO T) using python scipy (v. 1.3.0) and stats models packages (v. 0.24.2). Proteins that passed a Benjamini-Hochberg Q of 0.1 were considered significant [105].

### Functional analysis

KEGG Orthologs (KO) were extracted for each protein from the eggNOG-mapper results [106]. KEGG pathways, modules, and KO’s were separately quantified by the number of proteins associated with each, filtered by minimum count thresholds, and CLR transformed. These results were statistically analyzed by Two-Way ANOVA in R. *P*-values within each analysis where FDR was controlled by Benjamini-Hochberg procedure (Q < 0.25). Epsilon effect sizes were calculated with R package sjstats (v. 0.10.1) [107].

## Supplementary information

Supplemental Text and Figures

Supplemental Table S1

Supplemental Table S2

Supplemental Table S3

Supplemental Table S4

Supplemental Table S5

Supplemental Table S6

Supplemental Table S7

Supplemental Table S8

## Data Availability

All metaproteomics mass spectrometry measurements and identifications are available in massIVE, MSV000085110. All 16 S and whole genome sequencing data is available in SRA and all medium quality non-redundant genomes have been uploaded to genbank, PRJNA603829.
