# Supplementary material for: Morphine and high-fat diet differentially alter the gut microbiota composition and metabolic function in lean versus obese mice
Source: ISME Commun. 2022 Aug 5;2:66. doi: 10.1038/s43705-022-00131-6 (PMC9723762; doi:10.1038/s43705-022-00131-6)
Supplement: Supplementary file 1 — Supplemental Text and Figures [file 43705_2022_131_MOESM1_ESM.docx]

**Supplemental Material**

**Methods**

**Protein Extraction**

Between 8-50 mg of fecal material were placed in an Eppendorf tube. Approximately 200 ųL of 0.115 mm zirconium oxide beads were added to each tube, followed by 350-400 ųL of lysis buffer (2% SDS in 100mM Tris-HCL buffer) in each tube. Samples were lysed by bead beating in a Next Advance bead beater at speed 10 for 5 minutes. Samples were centrifuged at 21,000 g for 10 minutes. Next, 200 ųL of supernatant was pipetted for protein extraction, and the remainder (with beads) was used for DNA extraction. Protein extraction supernatant was heated at 85°C for 10 minutes, incubated with 25mM DTT for 20 minutes, and then incubated with 75 mM IAA for 20 minutes. Protein was then extracted using a chloroform methanol extraction protocol: addition of 800 ųL of methanol, 200 ųL of chloroform, and 600 ųL of water followed by 10 minutes of centrifugation at 21,000 g. Supernatant was disposed of and remaining pellet was washed using methanol. Dried pellet was reconstituted in sodium deoxycholate (SDC) solution made up of 4% SDC in 100 mM ammonium bicarbonate. Protein concentration was measured using bicinchoninic acid (BCA) assay. Each sample was adjusted to be 250 ųg of total protein in 500 ųL solution. Peptides were digested twice using a 1:75 (wt/wt) trypsin ratio, once for three hours and once overnight. After digested, solution was filtered to remove undigested peptides. Digestion reaction was quenched using 1% formic acid to lower pH, then solution was cleared with an ethyl acetate wash. Peptide concentration was determined via BCA assay. Peptides were then lyophilized/dried via SpeedVac and reconstituted in Solution A (95% LC-MS-grade H2O, 5% LC-MS-grade acetonitrile, 0.1% formic acid) at a concentration of 1 ųg/ųL.

**16S rRNA amplicon Sequencing and Analysis**

For 16S rRNA amplicon sequencing, the V4 hypervariable region of the 16S rRAN gene was amplified using universal primers derived from the original 515F and 806R primers (5′ GTGCCAGCMGCCGCGGTAA and 5’ GGACTACHVGGGTWTCTAA, respectively) (1) fused to Illumina sequencing adapters, following the procedure developed by (2). The reaction was supplemented with a 5% of a modified 515F primer that increases the coverage of TM7/Saccharibacteria (5′ GTGCCAGCMGCCGCGGTCA) (3). 12-nucleotide barcode sequences were incorporated into the second stage amplification reaction to enable sample multiplexing. The final amplicons were pooled, purified using Agencourt Ampure XP beads and quantified by Qubit. A pooled amplicon sample was sequenced (2 x 250 nt) on an Illumina MiSeq instrument (Illumina Inc, San Diego, CA) using a v2 500 cycle kit. Raw sequence reads were trimmed of the PCR primers/adaptors using cutadapt (<https://doi.org/10.14806/ej.17.1.200>) and joined using the QIIME script join_paired_ends.py.

Joined reads were processed using QIIME2 (v. 2019.1.0) (4). Reads were demultiplexed using the demux plug-in and denoised using the dada2 plug-in creating non-redundant amplicon sequence variants with a table of read counts per sample (5). A phylogenetic tree was created using the raxml-rapid-bootstrap plug-in with default settings (6). Based on the data summary, alpha diversity metrics Shannon, Simpson, and Faith were calculated using rarefaction at 50,000 reads (7, 8). Taxonomic assignment was performed against the SILVA SSU rRNA database (v. 132) (9, 10).

**Whole Genome Metagenomic Sequencing and Analysis**

Metagenomic sequencing was performed at the Sequencing Center of HudsonAlpha Institute (Huntsville, AL). Genomic DNA from 18 colon fecal samples was sheared and used to generate libraries that were sequenced (2 x 150 nt) on an Illumina NovaSeq instrument. The raw reads quality checked and trimmed using Trim Galore (v. 0.6.0) <https://www.bioinformatics.babraham.ac.uk/projects/trim_galore/>. Reads were then assembled with an aim to extract metagenome assembled genomes using a modified version of the procedure described in Olm et al (11). In brief, all 18 samples where co-assembled using MEGAHIT (1.1.3) with the parameters k-min set to 31 and k-step set to 10 (12). Mouse and human DNA was removed using bbsplit (v 38.31) against a masked human and mouse databases with the parameter minid set 0.95 similar to as described here <http://seqanswers.com/forums/showthread.php?t=42552>. Cleaned sequence reads were single sample assembled using idba_ud (1.1.3) with default parameters (13). Reads from all samples were mapped to all assemblies using bwa (v. 0.7.17-r1188) piped to samtools (v. 1.9) creating sorted bam files (14, 15). Subsequent bam files were used to bin contigs from all assemblies by coverage and tetranucleotide frequency using Metabat2 (v. 1.12.1) (16). Genomes were quality checked using checkM (v. 1.1.2) (17). Taxonomy was assigned to all genomes that passed the threshold >50% complete <25% contaminated using gtdb-tk (v. 0.2.2), which uses pplacer and ANI to classify genomes based on a database of over 150,000 genomes from NCBI and other sources (18, 19). Over 54% of genomes were >50% complete, and <10% contaminated out of the box, minimum standard for a medium quality genome so no further curation was done (20). Select genomes that were >50% complete >10% contaminated were refined using Anvi’o (v. 5.2.0) by tetranucleotide frequency clustering (21). Finally, all medium quality genomes, >50% complete and <10% contaminated were dereplicated using dRep (v. 2.2.3) at 99.5% ANI and 95% ANI to create non-redundant genome and species clusters respectively (11). For each group, the best quality non-redundant genome was selected as a representative genome. Protein sequences from the assemblies were extracted from the checkM result and annotated using emapper (v. 1.1.0.3-40-g41a8498) from the eggNOG database (22, 23). Abundance of each representative genome was calculated using the inner-quantile mean coverage (Q2Q3 mean coverage) of each genome as calculated by Anvi’o.

**Metaproteomic Measurement**

Colon extracted fecal samples were measured on a Q-Exactive Plus (ThermoFisher Scientific, Bremen, Germany) using an auto-sampled online two-dimensional chromatography system coupled to an electrospray emitter. Briefly, a three-phase back column was created using 150 um ID polyimide-coated fused silica containing 5cm of Aqua C18, 10cm of Luna strong cation exchange (SCX), and 5cm of Aqua C18 (Phenomenex, Torrance, CA). This frit was then connected to a 30cm C18 analytical column with an electrospray emitter at the end. A 12ųg of peptide solution, per sample from the previously detailed extraction method was loaded onto the back-column via an UltiMate 3000 HPLC auto sampler over a 30 minute load step, then 4 subsequent salt pulse steps of 35, 50, 100, 500 nano molar ammonium acetate were used to push peptides onto the analytical column. Peptides were then separated over a 150-minute reverse phase gradient, while being sprayed onto the mass spectrometer. A final 500 nano molar ammonium acetate salt pulse was used as a wash step prior to the load of the next sample. Peptides were detected using data dependent acquisition, with 5 peptides isolated for MS2 fragmentation per MS1. Peptides were fragmented using HCD fragmentation, and previously detected mass-to-charge ratios, where placed on a 30-minute exclusion list. Raw data was collected using Xcalibur (v. 4.1.31.9 ) and proprietary raw data format was converted to mzML using msconvert (v. 4.1.31.9 ) (24).

**Protein identification**

All prodigal gene calls extracted by running checkM on the medium quality representative genomes were extracted to create a protein database for proteomic analysis. The UniProt mouse proteome was added to this protein database, and all sequences were clustered at 98% identity using CD-HIT to remove redundancy (25). After, a list of common protein contaminants was added to the protein database. Protein identifications were conducted using the crux (v. 3.2.8aa66a2) framework (26). Initially an index of the protein database was created using tide-index with the settings: --missed-cleavages 2 --mods-spec C+57.02146,2M+15.9949 --clip-nterm-methionine T --decoy-format protein-reverse. Then an initial search was conducted using tide-search with a precursor window of 10 ppm. After this search using an in-house python script, a new protein database was created for each sample based on all proteins that were potentially matched to a spectrum. With these protein databases, each sample was searched against a sample specific database using tide-search with the following parameters: --compute-sp T --exact-p-value T --score-function both --precursor-window 10 --precursor-window-type ppm --num-threads 10 --mz-bin-width 1.0005079 (27, 28). Final search results were all submitted together to percolator for false discovery rate (FDR) calculation (29). For a protein to be identified, it needed to have at least one unique peptide identified with a peptide spectral match FDR of less than 1%. Protein FDR was also calculated, but not used in initial filtering. Retention time was extracted for all peptide spectral matches using an in-house script, and MS1 apex feature intensity was calculated for each peptide spectral match with a q-value < 0.01 using moFF (v. 2.0.3) (30). The moFF match between runs feature was specifically not used. Protein abundance for each sample was calculated by summing intensities for all unique MS1 features. Then all quantified proteins with a unique peptide were loaded onto a matrix. This matrix was log2 transformed, LOESS normalized, and mean centered using Inferno (v. 1.1.7234) (31). Proteins were also similarly quantified by spectral counting when appropriate.

**Richness and principal component comparison between conditions and omics methods**

Frequency of ASVs from QIIME2, inner-quantile of mean coverage of representative genomes from Anvi’o, genome summed spectral count from genome specific proteins, and individual protein intensity were all independently submitted to log transformation, loess normalization, and mean centering in Inferno (31). Subsequently missing values were imputed as zero, and principal components were calculated using base R (v. 3.6.3). Finally, PCA plots were rendered using ggplot2’s (v. 3.3.2) scatter plot feature. To determine richness of each measurement, the number of quantified ASV’s in each sample, genomes with an inner-quantile mean coverage >2, and number of proteins were counted per sample. Significant difference between conditions was calculated using the car (v.3.0-10) package Anova function and the TukeyHSD function in base R, and boxplots were rendered using ggplot2. Hierarchical clustering was conducted using the seaborn (v. 0.90) package in Python (v. 3.6.7)

**Evaluating newness of representative genome’s gene calls**

The newness of protein sequences taken from representative genomes was evaluated against a previously compiled mouse microbiome gene catalog (7/17/2015), nr (10/16/2019), environmental nr (9/28/2019), and UniParc (9/19/2019). All these databases were downloaded on October 16, 2019 (32, 33). A diamond blastp (v. 0.9.22) was then conducted between the protein database used for metaproteomic analysis and these four databases (34). Proteins that were not matched were recorded, and the top percent identity for each matched protein was also recorded.

**Differential protein analysis**

Normalized proteins intensities were filtered down to proteins that were quantified at least 3 times in a condition. These proteins were then submitted to serial t-tests between B6 control, and an experimental condition (B6 T, db/db S, db/db T, DIO S, DIO T). Using python scipy (v. 1.3.0) and stats models packages (v. 0.24.2), t-statistic, confidence interval, p-value, and Benjamini-Hochberg procedure was calculated for each comparison (35). Proteins that passed a Q of 0.1 were considered significantly different between two conditions. Prior to t-test calculation, missing values were imputed with a random number down shifted 2.8 standard deviations from the mean and within half a standard deviation. The goal was to avoid comparisons between zero and a large number, while ensuring that any situation where a protein was quantified in all samples in one condition and none in the other would declare that protein significant. All proteins in this set had a protein FDR below 5%. Species that had greater than 15 significant proteins in one condition were isolated for future analysis and rendered in a bubble plot using R. These species were also compared by number of proteins in a condition versus the inner quantile mean coverage of a representative genome of that species in the same conditions.

**Functional analysis**

KEGG Orthologs were extracted for each protein from the eggNOG-mapper results (36, 37). The KEGG ftp was used to connect KEGG orthologs to pathways, module, reactions, and BRITE hierarchies. For each grouping the number of quantified proteins per sample was counted. For Pathways, Modules, and BRITE hierarchies all groupings were counted in one matrix, and the count data was transformed and normalized by centered log ratio after filtering any category that did not have more than 4 proteins in at least one sample. The same was done for KO terms, but once for all KO terms with at least 4 proteins in a condition, and again for all KO terms with at least 7 proteins in a condition. The second filtering was done to help elevate more abundant KO terms from the noise. All of these results were statistically analyzed by Two-Way ANOVA using a for-loop in R. P-values within each analysis were stacked and FDR was controlled by Benjamini-Hochberg procedure (Q < 0.25). Epsilon effect sizes were calculated with R package sjstats (v. 0.10.1) (38).

**Cytokine Assays and Analysis**

Plasma samples collected as previously described and a third of each mouse’s colon was cryo grinded then the protein was extracted as previously described. Homogenates were analyzed for inflammatory factors using Milliplex MAP Mouse Cytokine/Chemokine Magnetic Bead Panel (Millipore Sigma, Burlington, MA) premixed 32-plex kit according to the protocol. All samples were 0.2 um filtered, and plasma volume was doubled (25 uL) to achieve detectable levels for most cytokines. Tissue supernatant volumes were based on protein concentration by NanoDrop readings of individual samples to achieve 15 ug total protein per well. Assay samples were aliquoted in triplicate; after protocol completion, plates were read on a Luminex analyzer.

Absolute quantitation in pg/mL for each cytokine (limit of detection 3.2 pg/mL) was achieved using Luminex software provided polynomial standard curves. Values below 3.2 pg/mL were imputed with a random number between 0 and 3.2 pg/mL. Exploratory data analysis using PLS-DA in the MixOmics R package was used to predict which cytokines were significantly more abundant in each line, and in morphine treatment (39) Two-way ANOVA followed by Tukey HSD was used to confirm the prediction.

**Urinary Metabolites**

Mouse urine collected during 24-hour metabolic cage duration was analyzed by LC-MS/MS on a Shimadzu LCMS-8040 in multiple reaction monitoring (MRM) mode for the following analyte transitions (m/z): morphine (285.70 > 201.00, 285.70 > 152.35), morphine-3-𝛽-D-glucuronide (462.20 > 286.15, 462.20 > 183.15, 462.20 > 58.20), hydromorphone (285.75 > 184.95, 285.75 > 157.00), hydromorphone-3-𝛽-D-glucuronide (462.20 > 286.10, 462.20 > 184.95), and normorphine (272.20 > 152.00, 272.20 > 165.00, 272.20 > 121.00). Spiked internal standards morphine-D6 (292.00 > 201.05, 292.00 > 152.95, 292.00 > 165.00) and morphine-3-𝛽-D-glucuronide-D6 (465.25 > 289.10, 465.25 > 201.10, 465.25 > 183.00) added to each sample at the same concentration for standardization and normalization. All standards obtained from Cerilliant (Round Rock, TX) were compiled into a stock solution used for serial dilution into aqueous urine matrix to achieve a nine-level calibration range: 10 to 5,000 ng/mL for morphine, hydromorphone, and normorphine; 50 to 25,000 ng/mL for morphine-3-𝛽-D-glucuronide and hydromorphone-3-𝛽-D-glucuronide. Urinary drug testing analysis confirmed morphine dosing of experimental treatment subjects in addition to hepatic drug metabolism among all three mouse lines.

**Description of supplementary tables**

**Supplementary Table 1:** Sample description and supporting data. A table that contains the experimental group characterization of each of the 18 mice as well as their respective morphine concentration in ng/mL, their plasma cytokine concentrations in pg/mL, and their colon tissue cytokine concentrations in pg/mL.

**Supplementary Table 2:** 16S rRNA feature table of detected amplicon sequence variants, with their sequences, their taxonomic assignment, and their counts per sample.

**Supplementary Table 3:** Descriptions of the representative genomes extracted from the metagenome that were uploaded to NCBI. Includes their genome and species group. Their bin identifier, their NCBI identifier, their assigned GTDB taxonomy, their assigned NCBI name, and their mean inner-quantile coverage per sample.

**Supplementary Table 4:** Table of proteins detected with at least one unique peptide. Contains their protein lever FDR, their assigned genome and species groups, their KEGG annotations, and their normalized abundances.

**Supplementary Table 5:** Results from protein differential abundance analysis. Contains protein identifiers, comparison, confidence interval, fold-change, p and q values, and log_2_ fold change.

**Supplementary Table 6:** Centered-log-ratio of number of proteins assigned to each KEGG pathway or module per sample, followed by two-way ANOVA analysis.

**Supplementary Table 7:** Centered-log-ratio of number of proteins assigned to each KEGG ortholog after filtering by at least 4 proteins detected in a condition followed by two-way ANOVA analysis.

**Supplementary Table 8:** Centered-log-ratio of number of proteins assigned to each KEGG ortholog after filtering by at least 7 proteins detected in a condition followed by two-way ANOVA analysis.

**Supplementary Figures**

**Figure S1**: Heatmaps depicting the ward clustering of (a) normalized inner quantile per genome mean coverage and (b) normalized read counts for 16S rRNA reads. (c) Boxplot of the percent amino acid identity of the top blastp hits between the microbial protein database created from this study’s metagenome and NR, UniPar, environmental NR, and a mouse catalog. (d) Family level LefSe analysis of the 16S results according to MicrobiomeAnalyst.


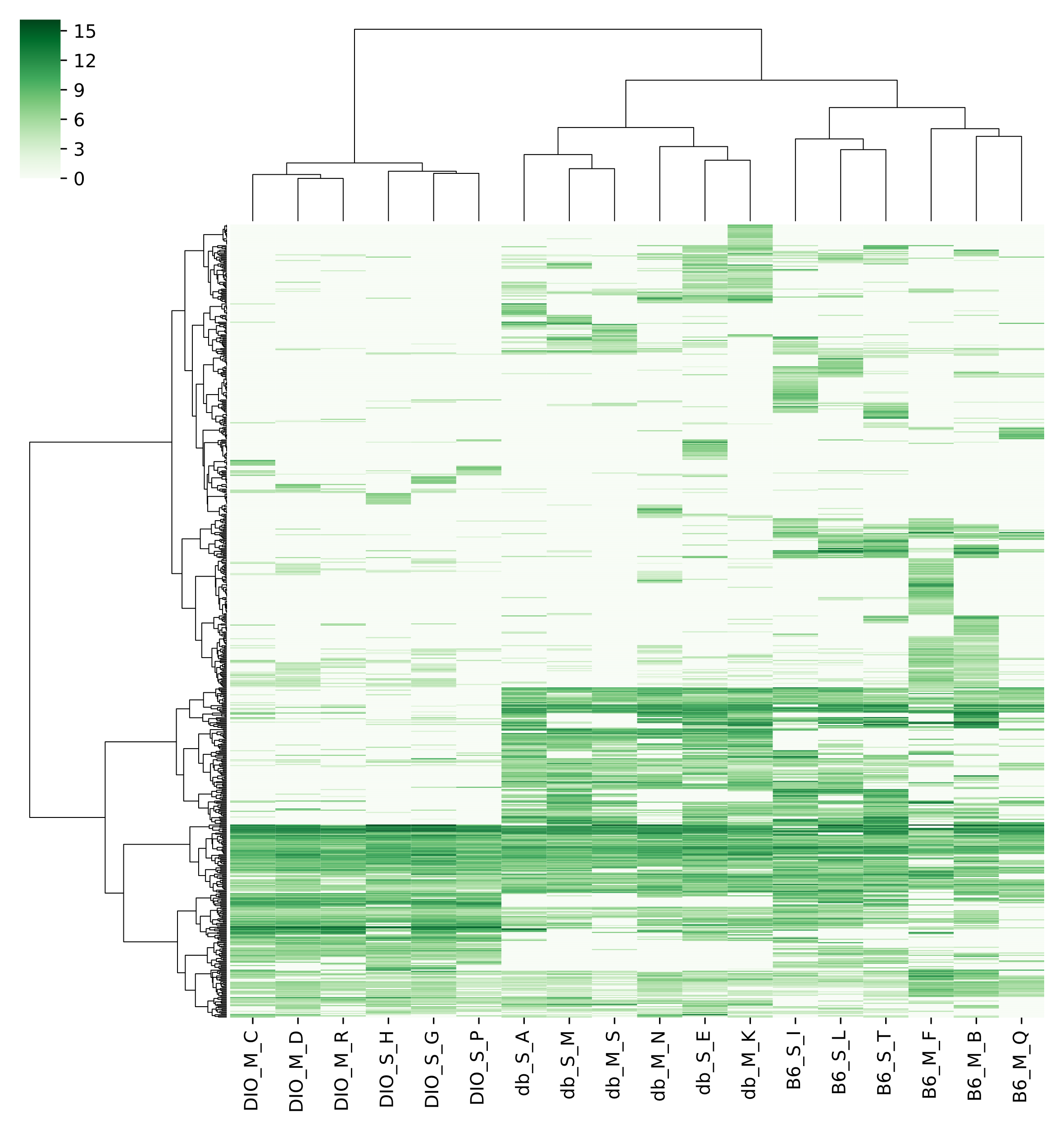

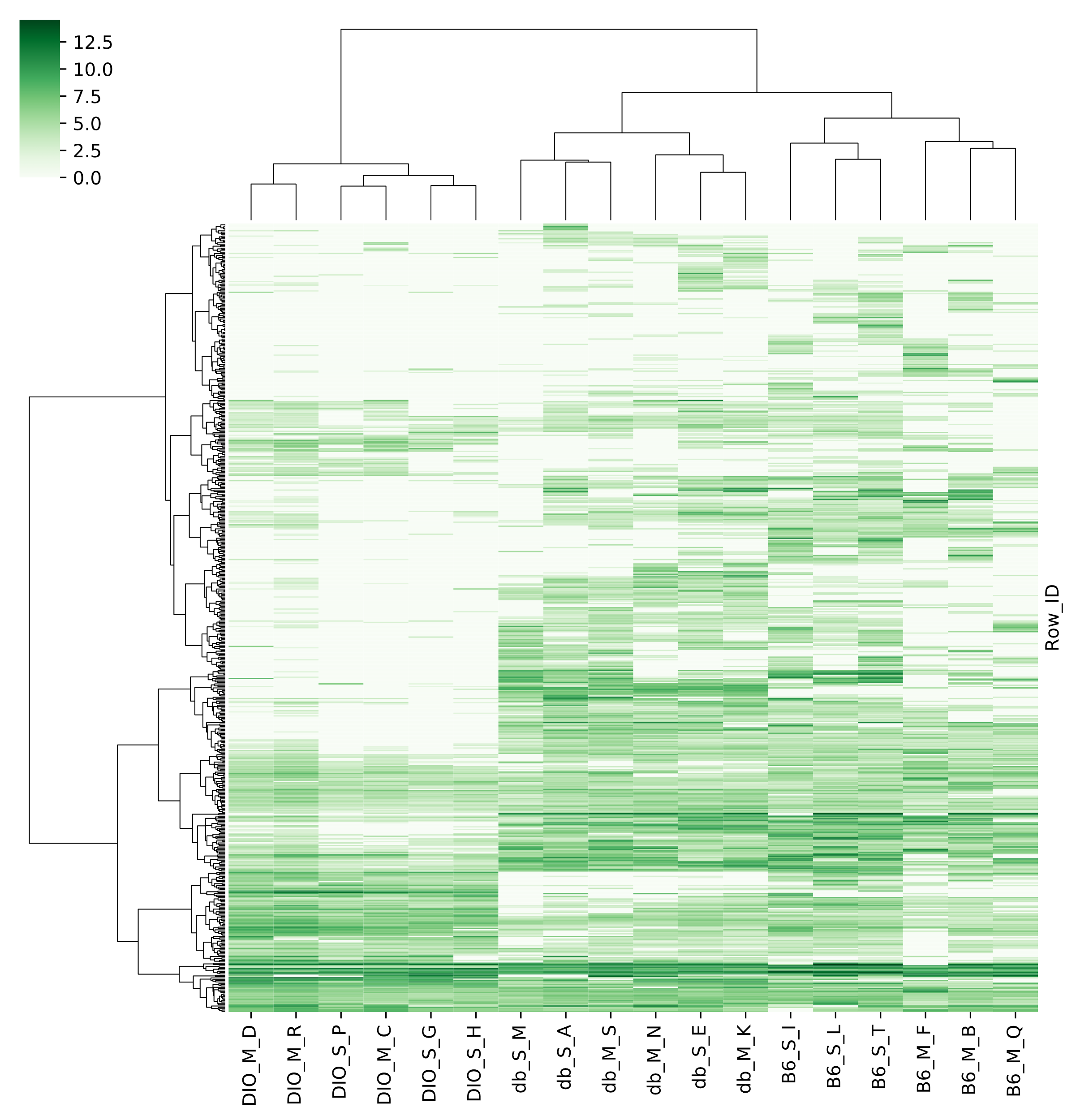

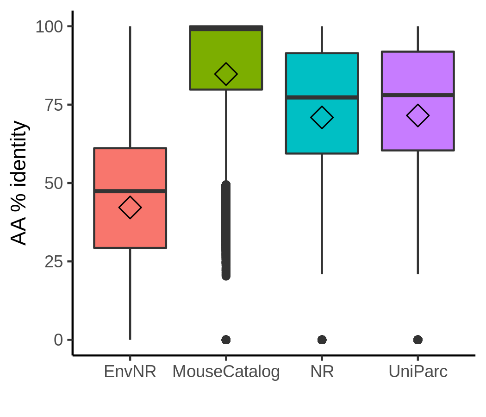


a.

b.

c.

d.


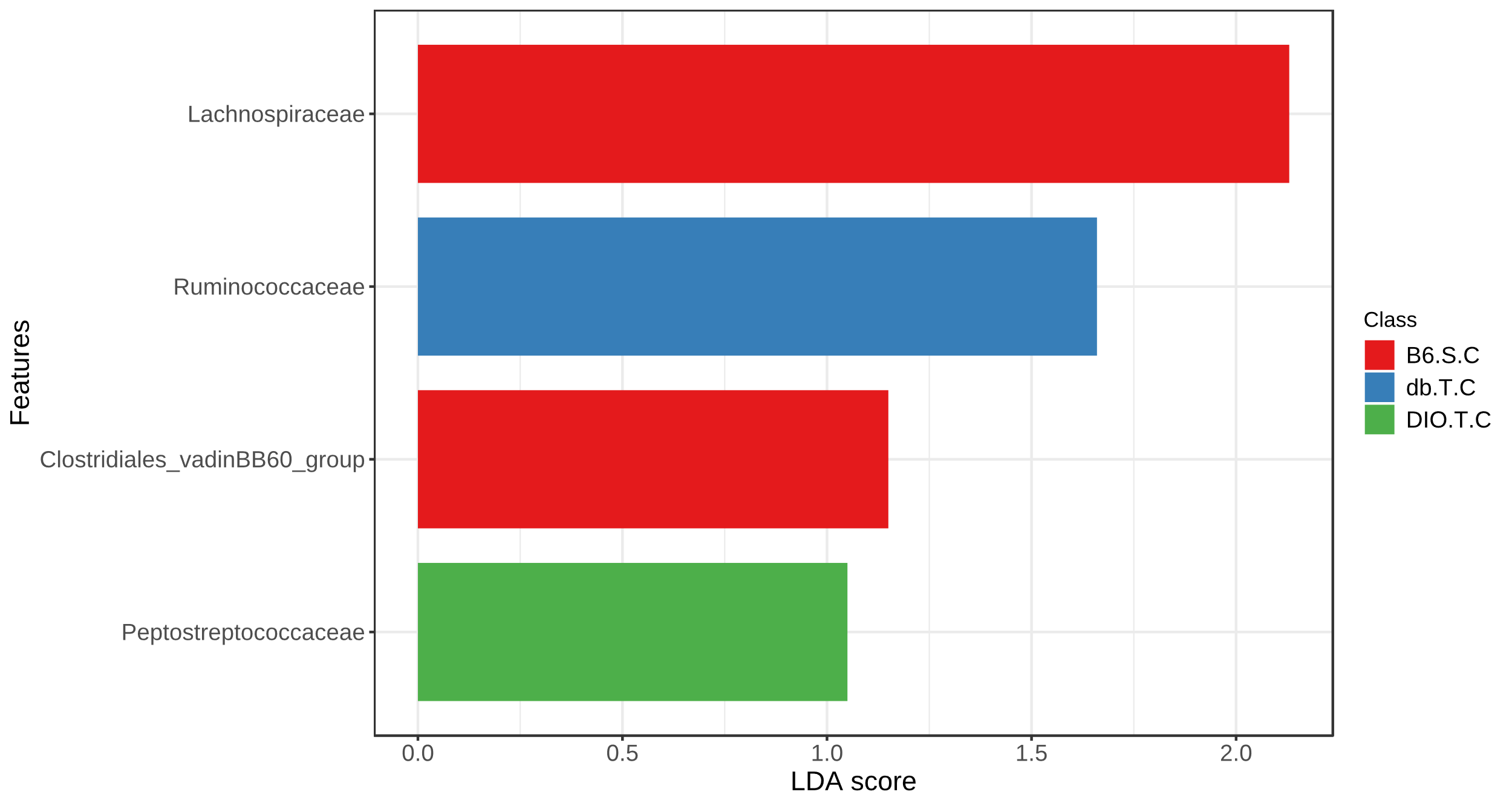


**Figure S2:** Stacked bar charts depicting percent abundance of phyla according to (a) 16S rRNA data and (b) inner quantile mean coverage of medium quality genomes. Note the two metrics are not directly comparable as they were analyzed using two different taxonomies, Silva versus for 16S data and Genome Taxonomy Database for metagenomics data.

**
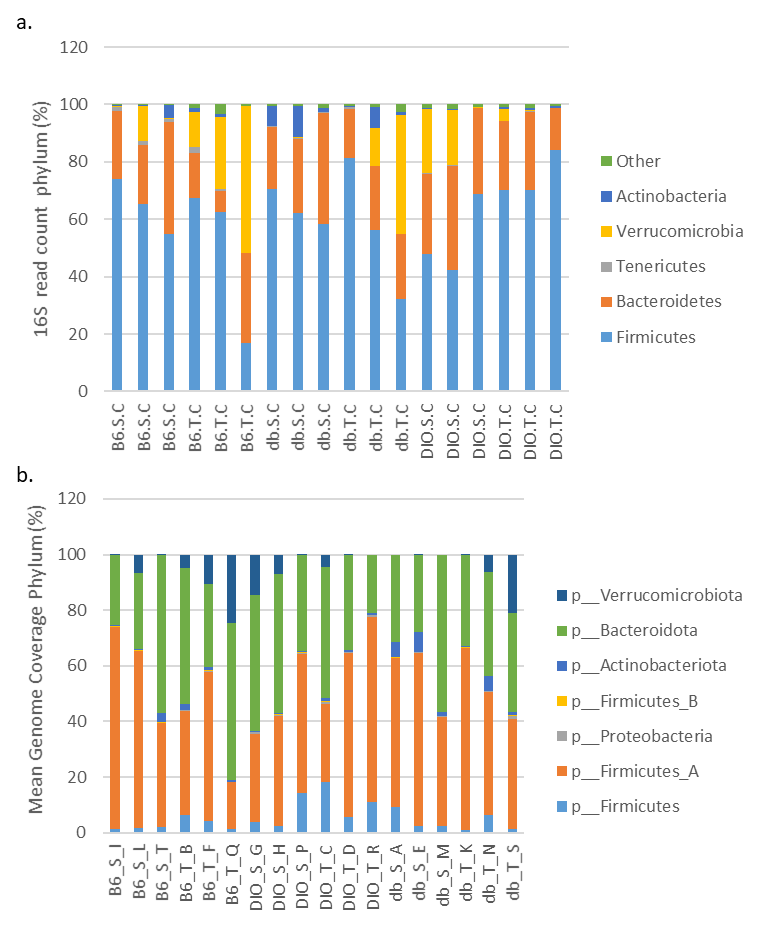
**

**Figure S3:** Boxplots depicting Faith, Shannon, and Simpson alpha diversity indices for 16S rRNA data.


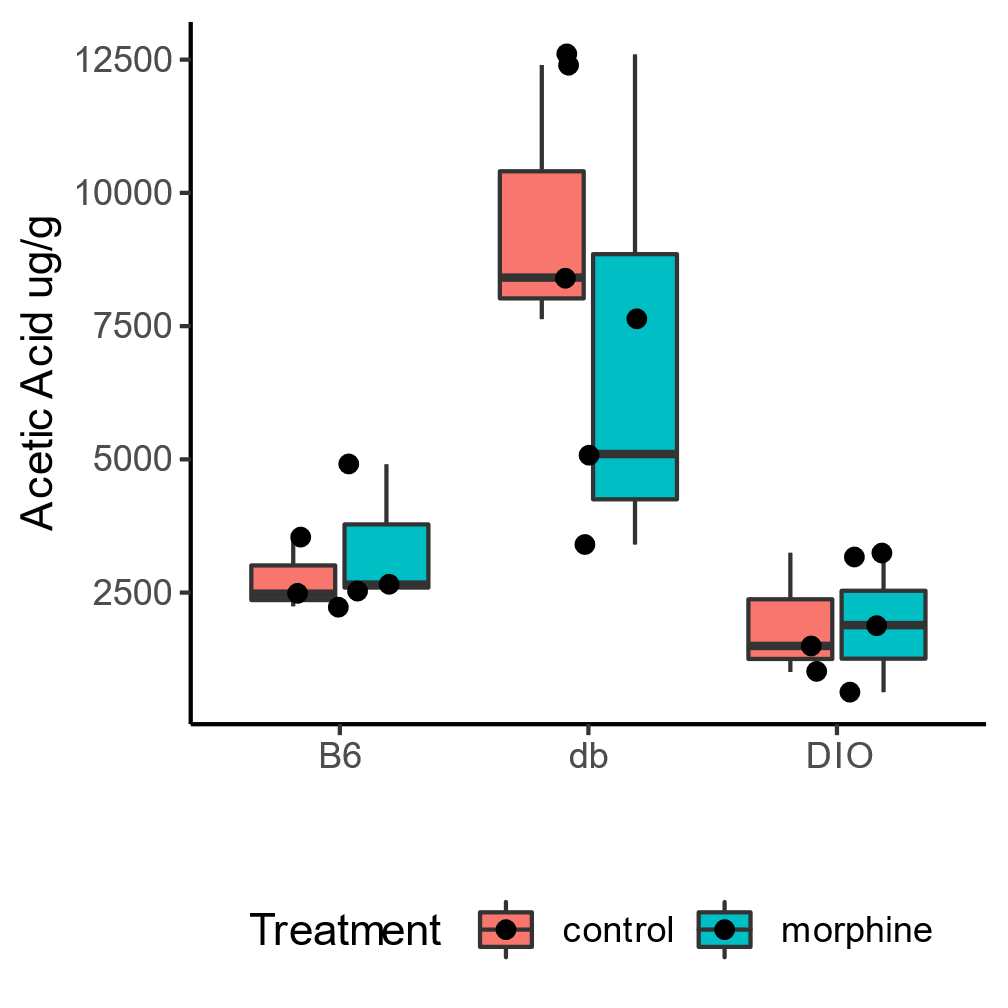


**Saline**


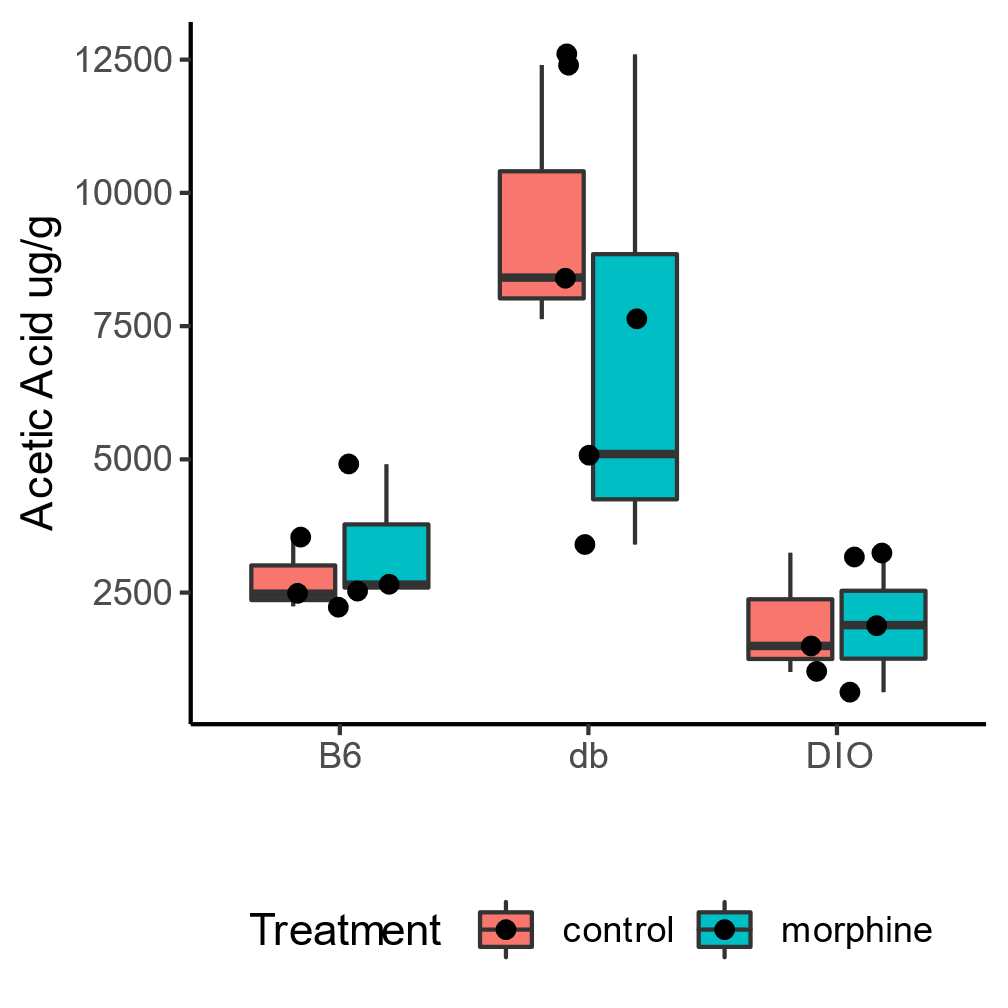


**Morphine**


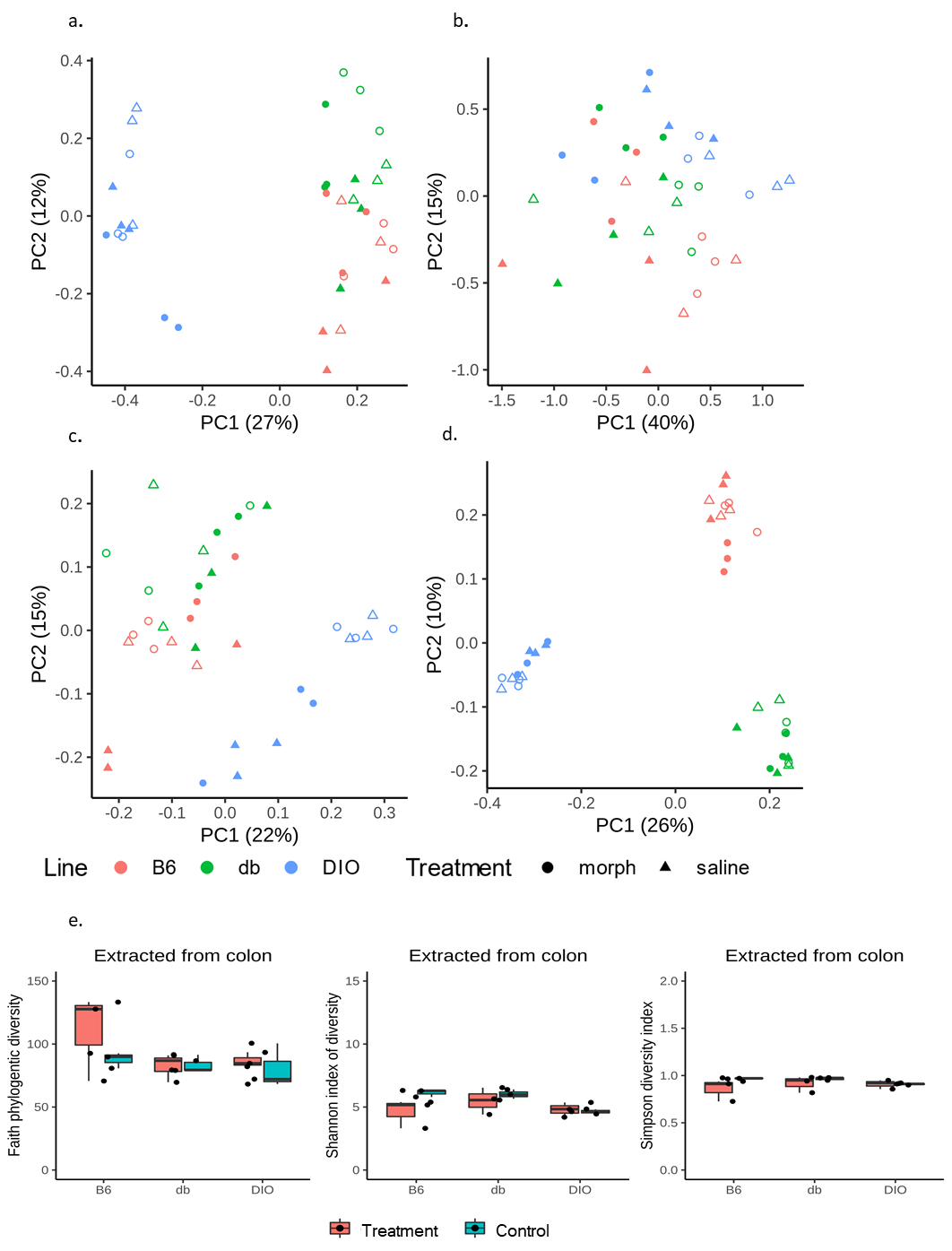


**Figure S4:** Boxplots depicting the number of proteins or inner-quantile mean coverage for each species that was overrepresented in the analysis of **Figure 3**.


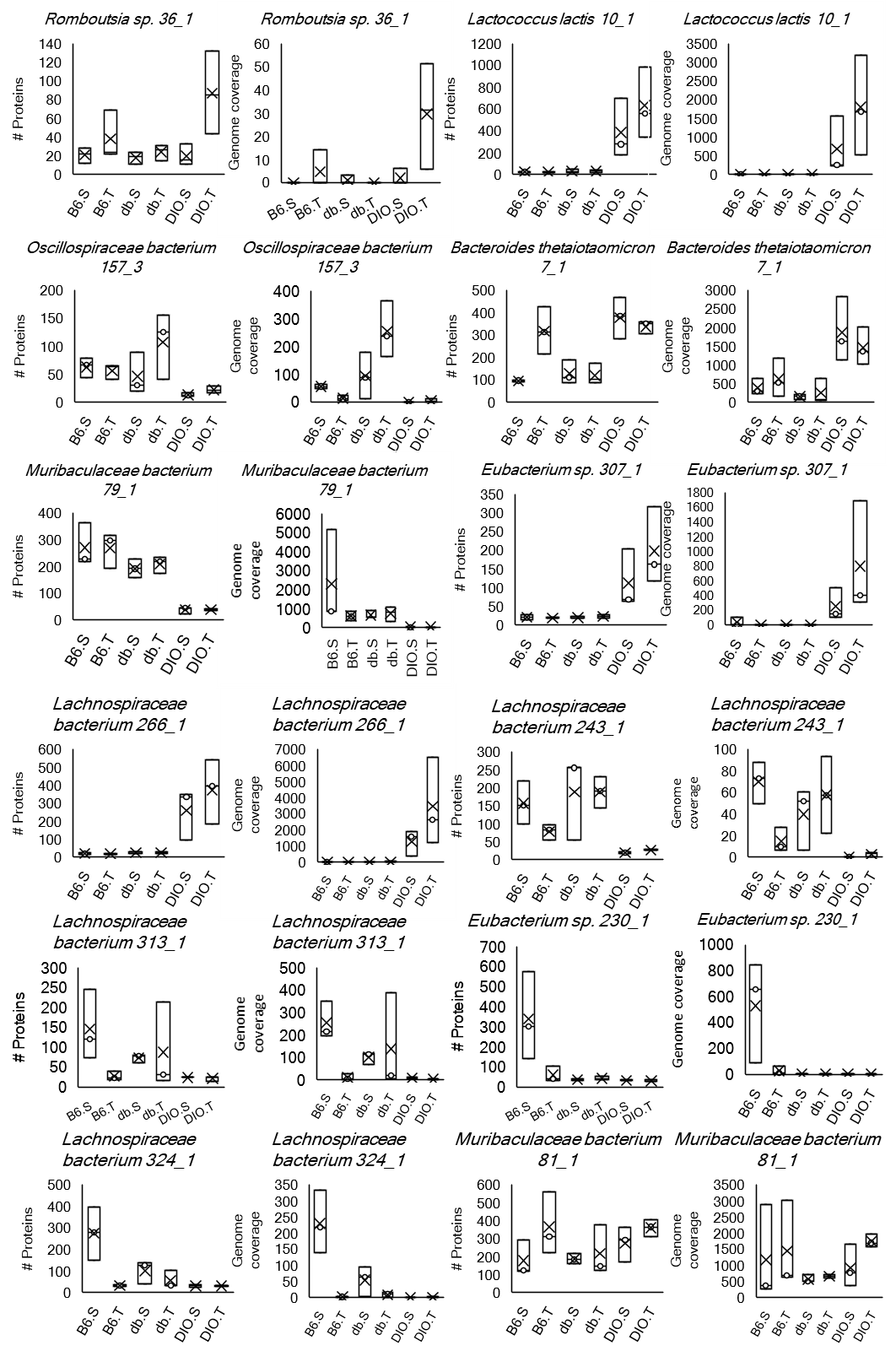


**Figure S5**: Additional enzymes beyond the ones presented in **Figure 5** that support the notion that high fat diet is pushing the microbiome towards the degradation of mucin. P-values are the result of TukeyHSD analysis after two-way ANOVA.

Xaa-pro dipeptidase


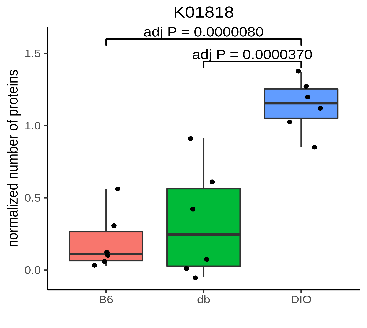

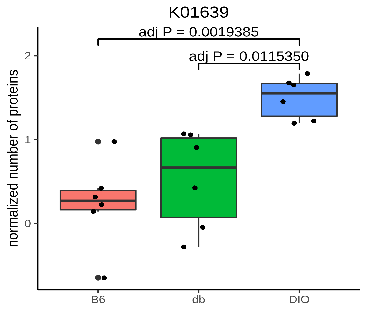

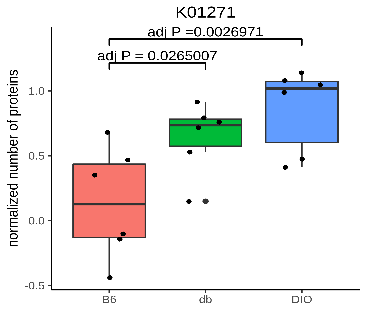


L-Fucose/D-arabinose

isomerase

Trimeric autotransporter adhesin


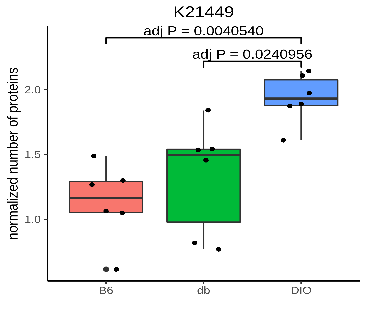

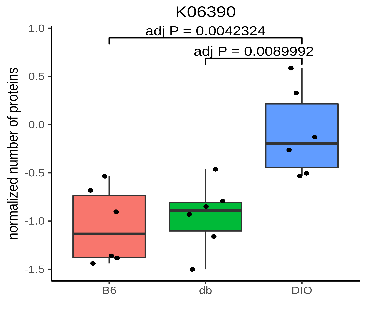


spoIIIAA


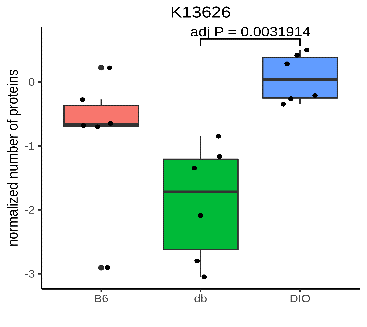


Flagellar assembly factor

Fucosidase


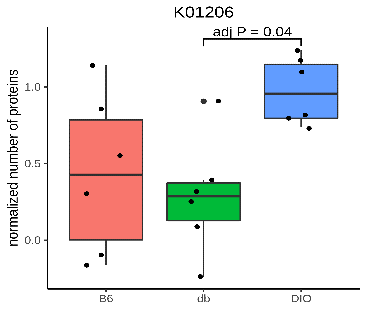


Sialidase


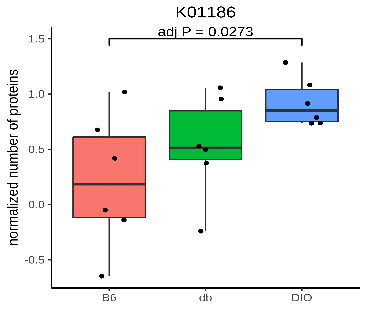


N-acetylneuraminate lyase

**Figure S6**: Cytokines significantly different between B6 saline and B6 morphine and different kinds of obesity. Significance was tested by welch t-test, when comparing B6 saline to B6 morphine and by ANOVA followed by Tukey HSD for comparisons between mouse lines. a-d represent cytokines different in plasma, while g-h represent cytokines different in tissue.


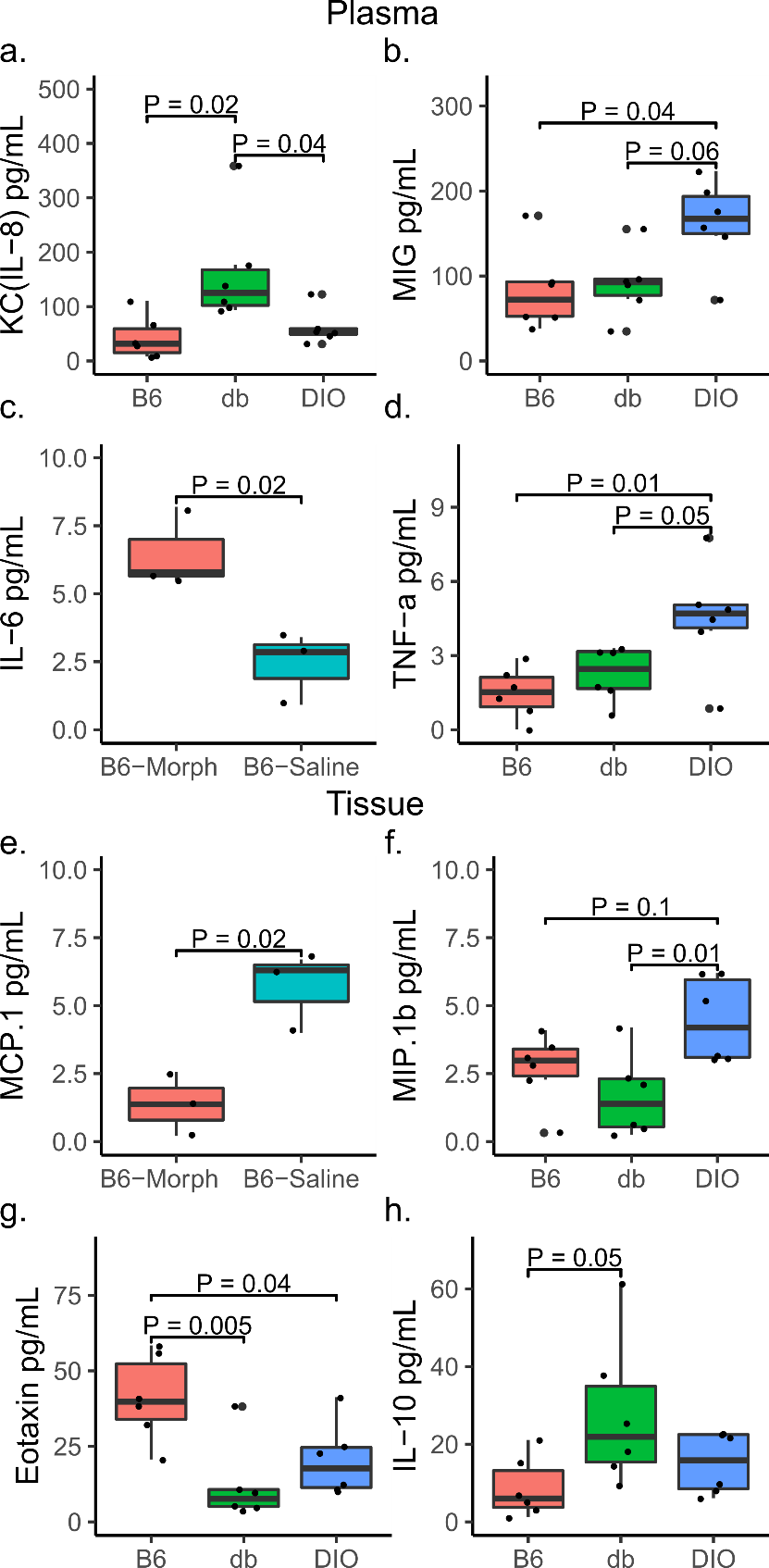


**References**

1. Bates ST, Berg-Lyons D, Caporaso JG, Walters WA, Knight R, Fierer N. Examining the global distribution of dominant archaeal populations in soil. The ISME Journal. 2011;5(5):908-17.

2. Lundberg DS, Yourstone S, Mieczkowski P, Jones CD, Dangl JL. Practical innovations for high-throughput amplicon sequencing. Nat Methods. 2013;10(10):999-1002.

3. Liang X, Whitham JM, Holwerda EK, Shao X, Tian L, Wu Y-W, et al. Development and characterization of stable anaerobic thermophilic methanogenic microbiomes fermenting switchgrass at decreasing residence times. Biotechnology for Biofuels. 2018;11(1):243.

4. Bolyen E, Rideout JR, Dillon MR, Bokulich NA, Abnet CC, Al-Ghalith GA, et al. Reproducible, interactive, scalable and extensible microbiome data science using QIIME 2. Nature Biotechnology. 2019;37(8):852-7.

5. Callahan BJ, McMurdie PJ, Rosen MJ, Han AW, Johnson AJA, Holmes SP. DADA2: High-resolution sample inference from Illumina amplicon data. Nature Methods. 2016;13(7):581-3.

6. Stamatakis A. RAxML version 8: a tool for phylogenetic analysis and post-analysis of large phylogenies. Bioinformatics. 2014;30(9):1312-3.

7. Faith DP. Conservation evaluation and phylogenetic diversity. Biological Conservation. 1992;61(1):1-10.

8. Lozupone CA, Hamady M, Kelley ST, Knight R. Quantitative and Qualitative β Diversity Measures Lead to Different Insights into Factors That Structure Microbial Communities. Applied and Environmental Microbiology. 2007;73(5):1576.

9. Pruesse E, Quast C, Knittel K, Fuchs BM, Ludwig W, Peplies J, et al. SILVA: a comprehensive online resource for quality checked and aligned ribosomal RNA sequence data compatible with ARB. Nucleic Acids Research. 2007;35(21):7188-96.

10. Bokulich NA, Kaehler BD, Rideout JR, Dillon M, Bolyen E, Knight R, et al. Optimizing taxonomic classification of marker-gene amplicon sequences with QIIME 2’s q2-feature-classifier plugin. Microbiome. 2018;6(1):90.

11. Olm MR, Brown CT, Brooks B, Banfield JF. dRep: a tool for fast and accurate genomic comparisons that enables improved genome recovery from metagenomes through de-replication. The Isme Journal. 2017;11:2864.

12. Li D, Liu C-M, Luo R, Sadakane K, Lam T-W. MEGAHIT: an ultra-fast single-node solution for large and complex metagenomics assembly via succinct de Bruijn graph. Bioinformatics. 2015;31(10):1674-6.

13. Peng Y, Leung HCM, Yiu SM, Chin FYL. IDBA-UD: a de novo assembler for single-cell and metagenomic sequencing data with highly uneven depth. Bioinformatics. 2012;28(11):1420-8.

14. Li H, Durbin R. Fast and accurate short read alignment with Burrows-Wheeler transform. Bioinformatics. 2009;25(14):1754-60.

15. Li H, Handsaker B, Wysoker A, Fennell T, Ruan J, Homer N, et al. The Sequence Alignment/Map format and SAMtools. Bioinformatics. 2009;25(16):2078-9.

16. Kang DD, Li F, Kirton E, Thomas A, Egan R, An H, et al. MetaBAT 2: an adaptive binning algorithm for robust and efficient genome reconstruction from metagenome assemblies. PeerJ. 2019;7:e7359.

17. Parks DH, Imelfort M, Skennerton CT, Hugenholtz P, Tyson GW. CheckM: assessing the quality of microbial genomes recovered from isolates, single cells, and metagenomes. Genome Research. 2015;25(7):1043-55.

18. Parks DH, Chuvochina M, Waite DW, Rinke C, Skarshewski A, Chaumeil P-A, et al. A standardized bacterial taxonomy based on genome phylogeny substantially revises the tree of life. Nature Biotechnology. 2018;36:996.

19. Chaumeil P-A, Mussig AJ, Hugenholtz P, Parks DH. GTDB-Tk: a toolkit to classify genomes with the Genome Taxonomy Database. Bioinformatics. 2019.

20. Bowers RM, Kyrpides NC, Stepanauskas R, Harmon-Smith M, Doud D, Reddy TBK, et al. Minimum information about a single amplified genome (MISAG) and a metagenome-assembled genome (MIMAG) of bacteria and archaea. Nature Biotechnology. 2017;35:725.

21. Eren AM, Esen ÖC, Quince C, Vineis JH, Morrison HG, Sogin ML, et al. Anvi’o: an advanced analysis and visualization platform for ‘omics data. PeerJ. 2015;3:e1319.

22. Huerta-Cepas J, Forslund K, Coelho LP, Szklarczyk D, Jensen LJ, von Mering C, et al. Fast Genome-Wide Functional Annotation through Orthology Assignment by eggNOG-Mapper. Molecular Biology and Evolution. 2017;34(8):2115-22.

23. Huerta-Cepas J, Szklarczyk D, Heller D, Hernández-Plaza A, Forslund SK, Cook H, et al. eggNOG 5.0: a hierarchical, functionally and phylogenetically annotated orthology resource based on 5090 organisms and 2502 viruses. Nucleic Acids Research. 2018;47(D1):D309-D14.

24. Chambers MC, Maclean B, Burke R, Amodei D, Ruderman DL, Neumann S, et al. A cross-platform toolkit for mass spectrometry and proteomics. Nat Biotechnol. 2012;30(10):918-20.

25. Fu L, Niu B, Zhu Z, Wu S, Li W. CD-HIT: accelerated for clustering the next-generation sequencing data. Bioinformatics. 2012;28(23):3150-2.

26. McIlwain S, Tamura K, Kertesz-Farkas A, Grant CE, Diament B, Frewen B, et al. Crux: Rapid Open Source Protein Tandem Mass Spectrometry Analysis. Journal of Proteome Research. 2014;13(10):4488-91.

27. Diament BJ, Noble WS. Faster SEQUEST Searching for Peptide Identification from Tandem Mass Spectra. Journal of Proteome Research. 2011;10(9):3871-9.

28. Howbert JJ, Noble WS. Computing exact p-values for a cross-correlation shotgun proteomics score function. Molecular & cellular proteomics : MCP. 2014;13(9):2467-79.

29. Käll L, Canterbury JD, Weston J, Noble WS, MacCoss MJ. Semi-supervised learning for peptide identification from shotgun proteomics datasets. Nature Methods. 2007;4(11):923-5.

30. Argentini A, Goeminne LJE, Verheggen K, Hulstaert N, Staes A, Clement L, et al. moFF: a robust and automated approach to extract peptide ion intensities. Nature Methods. 2016;13(12):964-6.

31. Polpitiya AD, Qian WJ, Jaitly N, Petyuk VA, Adkins JN, Camp DG, 2nd, et al. DAnTE: a statistical tool for quantitative analysis of -omics data. Bioinformatics. 2008;24(13):1556-8.

32. Xiao L, Feng Q, Liang S, Sonne SB, Xia Z, Qiu X, et al. A catalog of the mouse gut metagenome. Nature Biotechnology. 2015;33:1103.

33. The UniProt C. UniProt: a worldwide hub of protein knowledge. Nucleic Acids Research. 2018;47(D1):D506-D15.

34. Buchfink B, Xie C, Huson DH. Fast and sensitive protein alignment using DIAMOND. Nat Methods. 2015;12(1):59-60.

35. Benjamini Y, Hochberg Y. Controlling the False Discovery Rate: A Practical and Powerful Approach to Multiple Testing. Journal of the Royal Statistical Society: Series B (Methodological). 1995;57(1):289-300.

36. Tatusov RL, Galperin MY, Natale DA, Koonin EV. The COG database: a tool for genome-scale analysis of protein functions and evolution. Nucleic acids research. 2000;28(1):33-6.

37. Kanehisa M, Sato Y, Kawashima M, Furumichi M, Tanabe M. KEGG as a reference resource for gene and protein annotation. Nucleic Acids Research. 2016;44(D1):D457-D62.

38. Lüdecke D. sjstats: Statistical Functions for Regression Models. Zenodo2018.

39. Rohart F, Gautier B, Singh A, Lê Cao K-A. mixOmics: An R package for ‘omics feature selection and multiple data integration. PLOS Computational Biology. 2017;13(11):e1005752.
